# Predicting exposure concentrations of chemicals with a wide range of volatility and hydrophobicity in different multi-well plate set-ups

**DOI:** 10.1038/s41598-021-84109-9

**Published:** 2021-02-25

**Authors:** Julita Stadnicka-Michalak, Nadine Bramaz, René Schönenberger, Kristin Schirmer

**Affiliations:** 1 Eawag, Swiss Federal Institute of Aquatic Science and Technology, 8600 Dübendorf, Switzerland; 2 School of Architecture, Civil and Environmental Engineering, EPF Lausanne, 1015 Lausanne, Switzerland; 3 Department of Environmental Systems Science, ETH Zürich, 8092 Zürich, Switzerland

**Keywords:** Toxicology, Assay systems, High-throughput screening

## Abstract

Quantification of chemical toxicity in small-scale bioassays is challenging owing to small volumes used and extensive analytical resource needs. Yet, relying on nominal concentrations for effect determination maybe erroneous because loss processes can significantly reduce the actual exposure. Mechanistic models for predicting exposure concentrations based on distribution coefficients exist but require further validation with experimental data. Here we developed a complementary empirical model framework to predict chemical medium concentrations using different well-plate formats (24/48-well), plate covers (plastic lid, or additionally aluminum foil or adhesive foil), exposure volumes, and biological entities (fish, algal cells), focusing on the chemicals’ volatility and hydrophobicity as determinants. The type of plate cover and medium volume were identified as important drivers of volatile chemical loss, which could accurately be predicted by the framework. The model focusing on adhesive foil as cover was exemplary cross-validated and extrapolated to other set-ups, specifically 6-well plates with fish cells and 24-well plates with zebrafish embryos. Two case study model applications further demonstrated the utility of the empirical model framework for toxicity predictions. Thus, our approach can significantly improve the applicability of small-scale systems by providing accurate chemical concentrations in exposure media without resource- and time-intensive analytical measurements.

## Introduction

Multi-well (microtiter) polystyrene plates play an important role in high throughput screening (HTS) experiments as they allow testing of different chemical concentrations, effect types and/or experimental conditions in parallel^1^. Initially developed for enzyme assays and vertebrate cell culture-based tests, environmental toxicology is a field that increasingly makes use of such plates employing, e.g., fish cell lines^2–6^, algal populations^7^, microbial communities^8^ and fish embryos^6,9^. Cells and organisms are generally exposed in these plates to chemicals dissolved in aqueous exposure medium, and the selected effects are measured at predetermined time points. In line with the premise of rapid screening, the test chemical is commonly added once at the onset of exposure. Owing to the small medium volumes and extensive resource needs, exposure concentrations are rarely verified. This means that effective concentrations are derived based on the assumption that nominal concentrations adequately reflect exposure and that the chemical concentration is stable throughout the exposure duration. Yet, abiotic and/or biotic loss processes can negate this assumption and result, in fact, in an under-prediction of toxicity^10,11^. Knöbel et al.^12^ and Tanneberger et al.^13^, for example, reported up to two orders of magnitude differences between nominal and measured concentrations of very volatile (i.e. with Henry’s Law Constant logHLC > − 4 atm^1^ m^3^ mol^−1^) and/or very hydrophobic (i.e. with octanol–water partition coefficient logK_OW_ > 6) chemicals in 24-well plate bioassays with zebrafish embryos and the RTgill-W1 cell line, respectively.

To overcome this problem, modelling approaches have been established with the aim to predict the chemical concentration available for biological entities in multi-well polystyrene plates^14–17^. These models link physicochemical properties of a chemical, like its logK_OW_ and logHLC, with its bioavailable concentration in the exposure medium via partitioning. While Armitage et al.^14^ developed such a model to theoretically compare nominal and predicted chemical concentrations from assays presented in the ToxCast database, Fischer et al.^18^ fitted the partition coefficients between the polystyrene wells and the exposure medium to measured data and thereby predicted concentrations in the medium over time. Yet, while promising, these predictions were compared with measured samples derived in the absence of cells or organisms^18^. Additionally, both modelling approaches take into account chemical air–water partitioning but neglect the losses of chemical concentrations due to volatilization. Though different covering methods may reduce chemical loss, none of the thus far presented covers will completely eliminate it. Rather, covering methods by themselves may have an impact on the chemical distribution in well plate systems. For instance, Schreiber et al.^19^ noticed that the commonly used adhesive foil^2,9,12,13,20^ reduced the concentration of phenanthrene and phenanthridine in the exposure medium.

Given this background, we first tested the partitioning models by Armitage et al.^14^ and Fischer et al.^18^ for their ability to predict measured chemical exposure concentrations in well plate systems using published data^13,21,22^ (i) containing different biological entities; (ii) using different plate formats and (iii) being covered in different ways. We then set out to systematically explore the importance of the covering method and medium volume for the stability in the culture wells of chemicals with different volatility and hydrophobicity. We finally developed an empirical model to be able to predict losses for chemicals with a wide range of physico-chemical properties in different plate set-ups.

## Materials and methods

### Study overview

We applied a three-step strategy (Fig. S1). At first, the two existing models, Armitage et al.^14^ and Fischer et al.^18^ (from here on referred to as Armitage and Fischer model, respectively), were tested based on three publicly available datasets where chemical concentrations in exposure media were measured^13,21,22^. The results of this model analysis led us to perform experiments that specifically investigated the impact of different types of covers and media volumes on the losses, over time, of chemicals with differing hydrophobicity and volatility. Then, in a third step, we developed an empirical model approach to predict such chemical losses in multi-well plates for the three data sets initially used in step 1. The empirical model focussing on adhesive foil as most commonly used cover was subsequently extrapolated to other plate sizes, medium volumes or biology entities, and validated for chemicals with a wide range of volatility and hydrophobicity. In order to test whether the empirical model approach can improve toxicity predictions, the model was tested in two case studies. The first case study asked whether the model can explain the apparent differences in chemical toxicity determined when different medium volumes were used in an in vitro toxicity test. The second case study asked whether chemical medium concentrations, predicted by our empirical model, yielded in vitro effective concentrations (EC50 values) that were comparable to those derived from measured concentrations and thus equally predictive of in vivo acute fish toxicity.

### Implementation of Fischer and Armitage models

The approach described by Armitage et al.^14^ is a mass balance model that assumes instantaneous equilibrium partitioning between various phases of the in vitro test system. It accounts for the chemical distribution between the aqueous phase, headspace and cells/tissue. It also takes into consideration the presence of serum constituents and dissolved organic matter (DOM) as well as the chemical water solubility—all of which can influence the chemical distribution. However, the model does not account for chemical sorption to vessel walls. In the Armitage model, the freely dissolved aqueous concentration in the exposure medium (C_W_ in mol L^−1^) is estimated with the following equation:

1 $${\mathrm{C}}_{\mathrm{W}}=\frac{{\mathrm{M}}_{\mathrm{T}}}{{\mathrm{K}}_{\mathrm{AW}}{\mathrm{V}}_{\mathrm{A}}+{\mathrm{V}}_{\mathrm{W}}+{\mathrm{K}}_{\mathrm{SaW}}{\mathrm{V}}_{\mathrm{Sa}}+{\mathrm{K}}_{\mathrm{SIW}}{\mathrm{V}}_{\mathrm{SI}}+{\mathrm{K}}_{\mathrm{DW}}{\mathrm{V}}_{\mathrm{D}}+{\mathrm{K}}_{\mathrm{CW}}{\mathrm{V}}_{\mathrm{C}}}$$

where: M_T_ is the nominal chemical amount, K_AW_ is the air–water partition coefficient, V_A_ is the volume of head space, V_W_ is the volume of medium, K_SaW_ is the serum albumin-water partition coefficient, V_Sa_ is the volume of serum albumin, K_SIW_ is the serum lipid-water partition coefficient, V_SI_ is the volume of serum lipids, K_DW_ is the dissolved organic matter (DOM)—water partition coefficient, V_D_ is the volume of DOM, K_CW_ is the cell/tissue-water partition coefficient, and V_C_ is the volume of cell/tissue.

This approach requires the following chemical properties as the model input: chemical molecular weight, melting point, logK_OW_, logK_AW_ and water solubility. Table S1 presents the values of system parameters that had to be specified. The model has been implemented within the excel sheet provided by Armitage et al.^14^.

The Fischer model is a kinetic model, which focuses on chemical diffusion to the plastic, and can be applied to predict the depletion of organic chemicals from different bioassay media by sorption to various well plate formats^18^. Chemical concentration in exposure medium over time can be calculated from the polystyrene-water partition constant (K_PS/W_), which is estimated with the following equation:

2 $$\mathrm{Log }{\mathrm{K}}_{\mathrm{PS}/\mathrm{W}} =0.56\cdot {\mathrm{logK}}_{\mathrm{OW}}-0.05$$

This time-resolved approach requires fewer user inputs than the model by Armitage: chemical logK_OW_, dimensions of the used well plate, the lipid- and protein contents (to account for the serum presence in the medium) and the total volume of the exposure medium. In addition, even though the approach has been developed and verified only with the plates not containing any biological entity (i.e. cells or organisms), the authors stated that “the model can be extended for the chemical partitioning to cellular lipids and proteins by adding their respective volumes to the total volume of proteins and lipids in the system”^18^. Therefore within our study, the Fischer model has been applied without and with the extension accounting for the lipid volume, which was calculated from lipid fraction set to 5%, cell diameter set to 15 µm and cell number set to 300,000, according to the RTgill-W1 cell number used in Tanneberger et al.^13^. The model has been implemented within the excel sheet provided by Fischer et al.^18^.

### Origin of measured data used for testing Fischer and Armitage models

Three data sets (Fig. S1, Table S2), containing measured chemical concentrations, were used to test the Armitage and the Fischer models. None of them included serum in the exposure medium. From two databases (Tanneberger et al.^13^ and Dupraz et al.^21^), a few data points had to be excluded from the analysis as result of the quality check (see details in SI, p. 3).

Data from Tanneberger et al.^13^ were obtained from experiments performed at 19 °C in polystyrene (PS) plastic 24-well plates (Greiner Bio-One, Frickenhausen, Germany, cat. 656161) covered with an adhesive foil (VWR International GmbH, Darmstadt, Germany) and a PS plastic lid. Each well contained 2 mL of L15/ex medium^3^ with a chemical dissolved in DMSO (Dimethyl sulfoxide, final content: 0.5% v/v) and a monolayer of the RTgill-W1 cell line obtained from rainbow trout (*Oncorhynchus mykiss*) gills^23^ (seeded cell number = 300,000 per well). Data from Tanneberger et al. were originally used to derive chemical effective concentrations (EC50s) from sigmoidal concentration–response curves. Therefore, for each chemical, six different chemical concentrations were measured in the original study. For the purpose of our study, the ratios between the chemical medium concentration at the end of the experiment (i.e. 24 h) and at the beginning of the experiment (i.e. 0 h) were determined to quantify the chemical losses from the exposure medium in order to allow for cross-plate comparisons. The data used for the modelling, comprising 27 chemical data points, as well as chemical sources and physico-chemical properties, are presented in Table S2.

The data set based on Dupraz et al.^21^ included measured concentrations of 13 chemicals. Chemical medium concentrations were presented in 0.90 mL of culture medium with *Tisochrysis lutea* or *Skeletonema marinoi* microalga in transparent PS 48-well plates (Greiner Bio-One GmbH, cat. 677102). During the experiments, the plates were covered only with the plates’ PS plastic lid. The average values and standard deviations of the ratios between the chemical medium concentrations measured at the end (i.e. 96 h) and at the beginning (i.e. 0 h) of the experiment were calculated as explained above for different chemical concentrations. Thirteen chemical data points were available from this study as provided in Table S2 along with the chemicals’ properties.

Medium chemical concentrations measured in Schug et al.^22^ were determined in the in vitro system similarly as in Tanneberger et al.^13^ but with a monolayer of the rainbow trout gut cell line—RTgutGC^24^ (seeded cell number = 120,000 cells per well) and with PS well plates covered with a sheet of aluminium foil under the PS plastic lid. This foil did not contain any glue but was tightly wrapped over the plate. The exposure medium, solvent, exposure time and volumes used in the experiments were the same as in Tanneberger et al.^13^. The average values and standard deviations of the ratios between the chemical medium concentrations at the end (i.e. 24 h) and the beginning (i.e. 0 h) of the experiments were taken directly from the supplementary table provided by Schug et al.^22^. Sixteen chemical data points resulted from this study and are shown in Table S2.

### Testing different covering methods

Three different covering approaches were tested side-by-side for the purpose of the current study to investigate their impact on chemical losses from the exposure medium (L15/ex) not containing any serum: only PS plastic plate lid (as used in Dupraz et al.^21^; Greiner Bio-One, Frickenhausen, Germany), the adhesive foil (Thermo, sterile sealing tape, cat. 236366) + PS plastic lid (as used in Tanneberger et al.^13^) and the aluminium foil + PS plastic lid (as in Schug et al.^22^). Three chemicals were tested in 24-well plates with different medium volumes and without any biological material: difenoconazole, naphthalene and DTBP (2,4-di-tert-butylphenol), which cover logHLC values up to seven orders of magnitude difference (see Table S3). Sampling for chemical analysis was performed at the beginning of the experiment (time point 0 h), i.e. before the covers were placed (therefore there is one time 0 h for all three covers), as well as after 4 h and 24 h of exposure. A no more than 24 h exposure duration was judged to be appropriate in the context of these experiments because, on the one hand, very volatile chemicals (based on their logHLC) may not be detectable beyond this time frame while, on the other hand, steady-state medium concentrations are reached in this time frame even for chemicals with significantly greater hydrophobicity (based on logK_ow_) than relevant here^20^. In order to test the impact of the ratio between the air and medium volumes on the distribution of volatile chemicals, experiments with an adhesive or aluminium foil were carried out with 1 mL and 2 mL of the exposure medium. Chemical quantification of each of 144 samples (three independent experiments of 48 measured points with three technical replicates (averaged)) was done by a High Pressure Liquid Chromatograph equipped with a Fluorescence Detector (HP1200, HPLC-FLD, Agilent Technologies, Waldbronn, Germany). Details for sample preparation and chemical analytics are provided in SI, p.3.

### Development of the empirical model for in vitro systems covered with an adhesive foil

The model was developed in order to predict the chemical concentration in the exposure medium at the selected time point (usually the end of the experiment) as the fraction of the chemical concentration in the medium at the beginning of the experiment. The model equation and main assumptions are stated below.

#### Model assumptions

The computational model was developed based on the following assumptions:

- Chemical decrease in the exposure medium depends mostly on octanol–water partition coefficient (logK_OW_) and Henry’s Law Constant (logHLC).
- The relation between chemical parameters and medium concentration can be described with the sigmoid model.
- There is a threshold needed for logHLC from which this parameter matters regarding the chemical loss from the medium. As water logHLC is -6.38 (calculated based on water vapour pressure and density for 20 °C), the threshold (p3 from Eq. 3) can be expected to be characterized by a similar value.
- Chemical concentration in the exposure medium cannot be higher than that measured at the beginning of the experiment (i.e. at time point 0 h).
- Chemical concentration in the exposure medium cannot be lower than 0.

#### Model equation

The following equation was developed based on the above assumptions and the model’s p1-p4 parameters were fitted to the calibration data:

3 $$\frac{{{\text{C}}_{{{\text{medium}}}} \left( {\text{t}} \right)}}{{{\text{C}}_{{{\text{medium}}}} \left( {0\;{\text{h}}} \right)}} = \frac{1}{{1 + 10^{{({\text{p}}1 - {\text{Log Kow}} - {\text{p}}2 \cdot \max ({\text{LogHLC}} + {\text{p}}3,0)) \cdot {\text{p}}4)}} }}$$

where: C_medium_(t) and C_medium_(0 h) are the chemical concentration in the exposure medium at time t and 0 h (i.e. beginning of the experiment), respectively; p1, p2, p3 and p4 are fitted model parameters. In particular, p1 [−] is a value at x-axis at which the y-axis value is equal to 0.5, p2 [1/(atm^1^ m^3^ mol^−1^)] is the weight of LogHLC characteristic, p3 [atm^1^ m^3^ mol^−1^] is the threshold value for chemical volatility and p4 is the slope [−].

As the model has two independent variables (logK_OW_ and logHLC), in order to present the results in 2D plots, the rules from the logistic models were applied. Namely, after fitting all four parameters, values on the x-axis are equal to “logK_OW_ + p2·max(logHLC + p3, 0)” for each chemical.

#### Model implementation

The model and its analysis were implemented in MATLAB (MATLAB with Statistics and Optimization Toolboxes, Release 2016b, The MathWorks, Inc., Natick, Massachusetts, United States). The model equation was fitted to the calibration data by applying “fit” function using a non-linear least-squares method. Starting parameter values were as follows: p1 = 4 (based on prior knowledge and the fact that most of tested chemicals had logK_OW_ between 0 and 8), p2 = 1 atm^−1^ m^−3^mol^1^ (by setting 1 we assume that if logHLC is above the threshold value it is as important as hydrophobicity), p3 = 6 atm^1^ m^3^ mol^−1^ (water logHLC was estimated to − 6.38 atm^1^ m^3^ mol^−1^ but in literature also -5.61 atm^1^ m^3^ mol^−1^was used as the volatility threshold^13^; therefore, we used the mean value), p4 = − 1 (default slope for the logistic model); upper limit of perturbation was set to 0.1, the maximum number of function evaluations allowed was set to 600 and the maximum number of iterations allowed was set to 400. Both, the lower bound on the size of a step and the lower bound on the change in the value of the objective function during a step were set to 10^–6^.

### Origin of data used for the calibration and validation of the model for adhesive foil

The model was calibrated based on the data presented in Tanneberger et al.^13^ for three supporting reasons: the plates of this study were covered with the foil of interest, i.e. an adhesive foil; the exposure medium used did not contain serum which could influence the chemical bioavailability^25–27^; and a wide range of chemicals characterized by different physico-chemical properties were covered. Thus, the calibration data consist of 27 different organic chemicals with logK_OW_ in the range between − 4.15 and 6.5 and logHLC in the range between − 12.1 and − 1.3 atm^1^ m^3^ mol^−1^. Details about the data are provided in Table S3.

The validation data originate from 40 experiments independent of the study that provided the calibration data, performed either on cell lines or zebrafish embryos. Data were obtained from our laboratory as part of other projects, some of which have already been published in other contexts in Stadnicka-Michalak et al.^20^ and Knöbel et al.^12^ (see details in SI, Table S3). It is important to highlight though that chemical concentrations from Stadnicka-Michalak et al.^20^ were measured based on radioactivity (^14^C-labelled compounds). Thus, opposite to all other experiments, chemical quantification accounts for both, potentially formed biotransformation products and the respective parent compound. Regarding the tests with zebrafish embryos, they were carried out for 48 h (compared to 24 h for cell lines). However, in our previous study^20^, we have noticed that steady-state conditions were generally reached much before 24 h of exposure. Thus, notable differences between the chemical concentration in the medium after 24 h and 48 h of exposure were not expected.

Of the validation data set, fifteen chemicals, different from those used for the model calibration, have been measured in exactly the same system as the calibration data (i.e. 24-well plate, 2 mL of L15/ex medium, RTgill-W1 cell line). Four chemicals have been tested in 24-well plates with 2 mL of cell culture medium (experiments with both L15/ex and L15 with 5% FBS media) but without cells. Model extrapolations to different exposure systems have been validated based on data for L15 medium with 5% FBS, for 6-well plates as well as for experiments with zebrafish embryos.

### Cross-validation of the model for adhesive foil

The model for in vitro systems covered with an adhesive foil was cross-validated based on the k-fold approach^28^ (fivefold) using MATLAB. Namely, the calibration and validation data, described above, were merged and 80% of the data were randomly selected and used as a model calibration set, while the remaining 20% of the data were used as the validation set. The data selection as well as the model calibration and validation were repeated 100,000 times and the statistical evaluation of the model was reported (for statistical methods see “Materials and methods”, “Statistical analyses”).

### Model extrapolation to other experimental set-ups

The validated model for 24-well plates, covered with an adhesive foil and containing L15/ex medium (without serum) and a confluent layer of RTgill-W1 cells, was extrapolated to other experimental set-ups. To do so, data of the distribution of several chemicals in a plastic well from Stadnicka-Michalak et al.^20^ were used to deliver partitioning coefficients between different compartments (i.e. medium, cells, plastic and headspace, in Fig. S2). These partition coefficients were then normalized based on the plastic surface, cell surface and volume, and the air volume (Table S4, example of chemical partitioning in Fig. S3).

By using the normalized partitioning between different compartments, it was possible to calculate the chemical distribution in different systems. For instance, in the system with 1 mL of L15/ex medium instead of 2 mL, the plastic surface touching the medium decreases two-fold, cell and foil surfaces stay the same and headspace volume increases 1.76-fold (calculated as V_W_—medium volume, see Fig. S2). The conversions between different well plate sizes integrated the mass balance equations with the reported experimental data and were applied as described in detail in Fischer et al.^18^. The well parameters for 24-well plates containing RTgill-W1 cells and different volumes of the exposure medium, or the zebrafish embryo and 2 mL medium, and for 6-well plates with the RTgill-W1 cell monolayer were used for the model extrapolations, and are presented in Table S3.

### Model applicability for improving toxicity predictions

In order to test whether the newly developed empirical models can help to improve the assessment of effective concentrations in the absence of chemical analysis, the model for adhesive foil was applied to two additional toxicity data sets.

In the first scenario, experiments with 27 organic chemicals from Tanneberger et al.^13^ were repeated in the same experimental set-up but with 1 mL of exposure medium instead of 2 mL as used in Tanneberger et al.^13^. Then, the EC50 values were determined based on nominal concentrations for each chemical tested with 1 mL and 2 mL of exposure medium. In addition, the developed empirical model was used in order to predict chemical concentrations at the end (i.e. at 24 h) of the exposure as a fraction of the respective nominal concentrations. Then, the geometric mean of the starting (nominal) and concluding (modelled 24 h) medium concentrations was calculated, as suggested by Tanneberger et al.^13^, and the nominal EC50s were converted to EC50s based on such predicted concentrations. If the volume of the exposure medium does not play a role in the toxicity experiments, the ratio of EC50(1 mL)/EC50(2 mL) would equal 1. However, if the chemical evaporates and/or binds to plastic surfaces, less of it would stay in the medium, and therefore be available for cells to be taken up. More medium means more grams of a chemical added to the system and therefore also more chemical is available for cells despite partial evaporation/plastic binding processes. As a consequence, in the nominal exposure concentration-based experiments with 1 mL medium, the EC50s could be expected to be higher than EC50s determined with 2 mL medium, and hence, the EC50(1 mL)/EC50(2 mL) ratio would be higher than 1. In theory, the predicted EC50s should account for this difference in the medium volume, and therefore, the EC50(1 mL)/EC50(2 mL) ratio should not significantly deviate from 1.

The second data set was published by Natsch et al.^29^ and included the comparison between fish LC50 data of 38 fragrances with the respective measured in vitro (RTgill-W1) EC50s derived with the Presto Blue dye which is a measure of the mitochondrial respiration/metabolic activity^30^. The experiments were performed in 24-well plates with 2 mL of exposure medium and a monolayer of the RTgill-W1 cell line. The plates were covered with an adhesive foil and the plate plastic lid. The arithmetic mean of the measured starting and concluding chemical concentrations in the exposure medium were used in order to determine in vitro EC50s and compare them with respective fish LC50s. Therefore, in our study we have tested whether using predicted medium concentrations (as described in the paragraph above) instead of the measured ones would result in the same agreement between the in vitro EC50 and fish LC50 data.

### Statistical analyses

Regarding the statistics for goodness of fit, the following measures have been provided: sum of squares due to error (SSE), R^2^ (coefficient of determination), degrees of freedom in the error and degree-of-freedom adjusted coefficient of determination, as well as the root mean squared error (RMSE). In addition, F-test, two-way ANOVA and Tukey test^31^ were performed in order to analyze the data.

Coefficient of determination (R^2^), here, refers to the square of the correlation coefficient between measured and modeled values, and compares the model behavior with the data characteristic (Eq. 4, based on the FOCUS guidance document^32^). According to this method, if R^2^ is closer to 1, the model fits the measured internal concentrations better.

4 $${\mathrm{R}}^{2}={\left(\frac{\sum_{\mathrm{i}=1}^{\mathrm{n}}\left({\mathrm{P}}_{\mathrm{i}}-\stackrel{-}{\mathrm{P}}\right)\left({\mathrm{O}}_{\mathrm{i}}-\stackrel{-}{\mathrm{O}}\right)}{\sqrt{\sum_{\mathrm{i}=1}^{\mathrm{n}}{\left({\mathrm{P}}_{\mathrm{i}}-\stackrel{-}{\mathrm{P}}\right)}^{2}{\left({\mathrm{O}}_{\mathrm{i}}-\stackrel{-}{\mathrm{O}}\right)}^{2}}}\right)}^{2}$$

where: n is the total number of paired observations (P, O), P_i_ is the ith value of the predicted internal concentration (with i = 1,2,…,n), O_i_ is the ith value of the measured internal concentration (with i = 1,2,…,n), ${\overline{\text{P}}}$ is the mean of all values for predicted internal concentrations and ${\overline{\text{O}}}$ is the mean of all values for measured internal concentrations.

RMSE (root mean square error) is one of the standard statistical metrics used to measure the model performance by estimating its errors^33^, and can be determined as follows:

5 $$\mathrm{RMSE}=\sqrt{\frac{\sum \left({\mathrm{residual}}^{2}\right)}{\mathrm{n}-1}}$$

where: residual is the vertical distance (in Y units) of the point from the fit line or curve, and n is the number of residuals.

## Results

### Existing models vs. measured data

**Figure 1:**
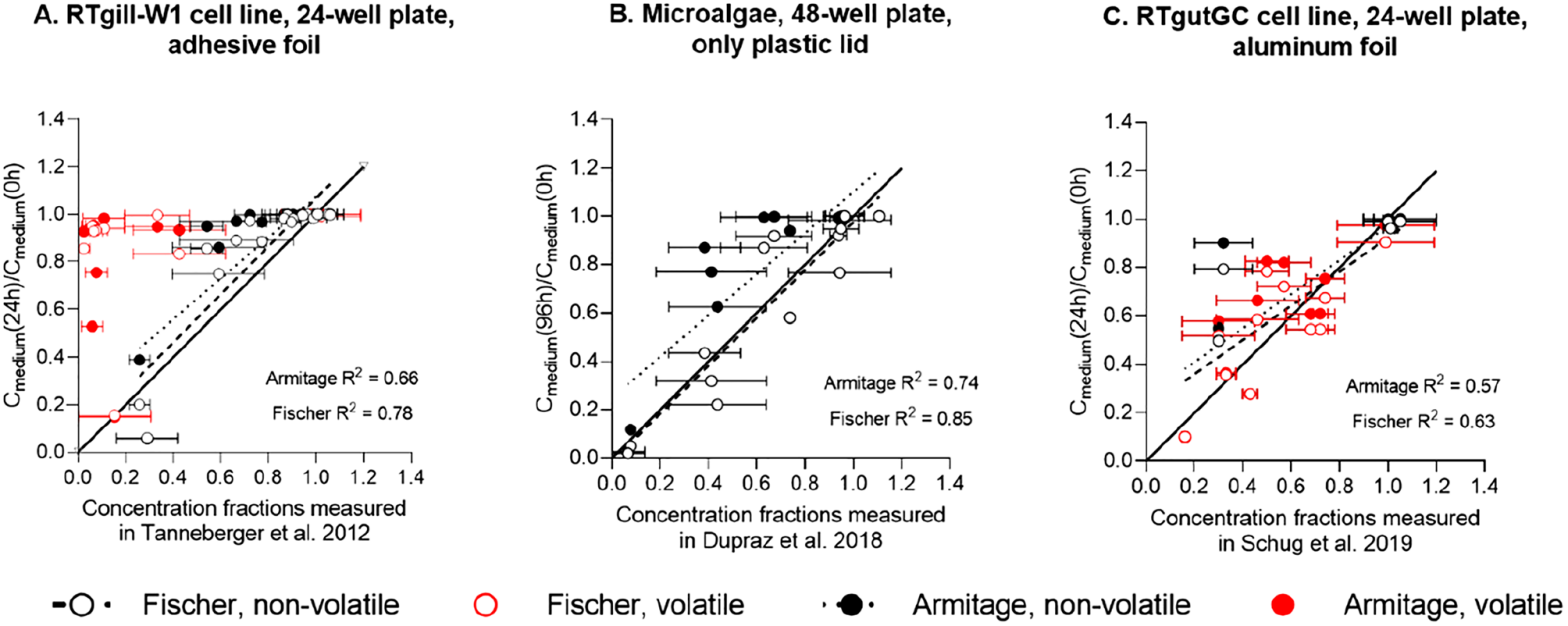
Comparison of measured and predicted chemical concentration fractions in exposure media, with predictions based on the Armitage (filled circles) and Fischer (empty circles) models. (**A**) 24-well plates covered with an adhesive foil (24 h RTgill-W1 cell line exposure, measured data from Tanneberger et al.^13^), (**B**) 48-well plates covered with plastic lid (96 h microalgae exposure, measured data from Dupraz et al.^21^) and (**C**) 24-well plates covered with aluminium foil (24 h RTgutGC exposure, measured data from Schug et al.^22^). Red symbols represent volatile chemicals (i.e. logHLC > − 5.61, according to Tanneberger et al.^13^). Solid line is a 1:1 correlation, dotted and dashed lines represent the regressions according to predictions by the Armitage et al.^14^ and Fischer et al.^18^ models, respectively (for adhesive foil the regressions of only non-volatile chemicals are shown).

The models by Armitage et al.^14^ and Fischer et al.^18^ overall predicted higher chemical concentrations in exposure medium than measured (Fig. 1). Yet, the extent of overprediction differed depending on the physico-chemical properties of the test chemicals and on the methods used to cover the plates. For well plates covered with an adhesive foil and for non-volatile chemicals (i.e. logHLC < − 5.61, as defined in Tanneberger et al.^13^; Fig. 1A, black circles), the Fischer model outperformed the Armitage approach (Table 1). The Fischer model has explicitly not been developed for volatile chemicals but the Armitage model could not predict concentrations of volatile chemicals correctly either (Fig. 1A, red circles). For 48-well plates covered with plastic lid (Fig. 1B), the Fischer model also outperformed the Armitage approach (all chemicals were non-volatile). For 24-well plates covered with aluminium foil (Fig. 1C), due to the insufficient number of non-volatile chemicals (five, of which three did not present any loss over time regarding their medium concentration), volatile and non-volatile chemicals were additionally analysed together (R^2^ on Fig. 1C). While there was a significant improvement regarding the models’ prediction of medium concentrations of volatile chemicals with aluminium foil as cover, concentrations were not as well predicted as for non-volatile chemicals (Table 1, studies of Tanneberger et al.^13^ and Dupraz et al.^21^). These results indicate that the models’ performance depended on the chemical volatility (Table 1) and, linked to this, the covering method. The models’ agreement with the measured data was very similar for different cell lines and non-volatile chemicals (see Table 1—R^2^ values for different cell lines and respective models), and therefore indicated that the covering method might play a more important role than the biological entity used. For this reason, the impact of covering methods on the concentration loss of chemicals with different volatility was further tested.

**Table 1:** Analysis of the Fischer and Armitage models based on the literature data.

Endpoint	Model	Tanneberger et al.^13^		Dupraz et al.^21^	Schug et al.^22^		
		Non-volatile^c^	Volatile^c^	Non-volatile^c^	Non-vol.^c^	Volat.^c^	All^d^
R^2^	Armitage^14^	0.66	0.15	0.74	0.61*	0.59	0.57
	Fischer^18^	0.78	0.07	0.85	0.77*	0.59	0.63
RMSE^a^	Armitage^14^	0.15	0.26	0.18	0.14*	0.18	0.19
	Fischer^18^	0.13	0.25	0.15	0.12*	0.15	0.15
Concentration overprediction^b^	Armitage^14^	81%		85%	50%		
	Fischer^18^	85%		31%	44%		

*Only five data points available and three of them did not present any loss in the medium

^a^Root Mean Square Error.

^b^States for how many chemicals (in %) model predicted higher medium concentration than it was measured.

^c^Volatile chemicals: logHLC > − 5.61, according to Tanneberger et al.^13^.

^d^Due to insufficient number of non-volatile chemicals (five, of which three did not present any loss over time regarding their medium concentration, see*), volatile and non-volatile chemicals were also analysed together.

### Importance of the covering method

**Figure 2:**
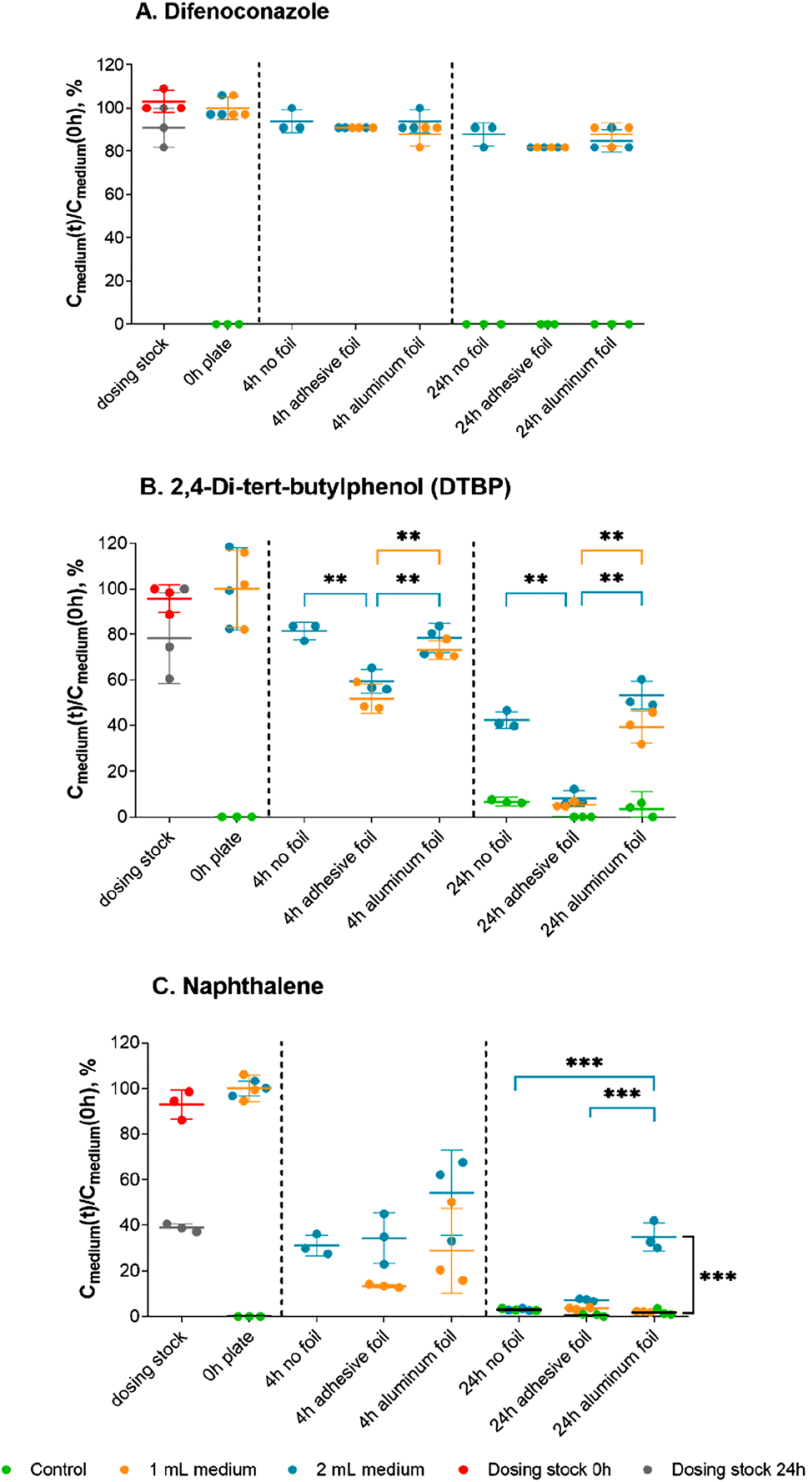
Impact of the covering method on losses of chemicals with different volatility. On the y-axes, the chemical concentration measured in the exposure medium at time t is shown as the percentage of the concentration at time point 0 h. (**A**) non-volatile, hydrophobic (logHLC = − 11.05, logK_OW_ = 4.3) difenoconazole, (**B**) semi-volatile, hydrophobic (logHLC = − 5.43, logK_OW_ = 5.19) 2,4-di-tert-butylphenol, (**C**) volatile, less hydrophobic (logHLC = − 3.36, logK_OW_ = 3.3) naphthalene. Stars represent significantly different groups according to two-way ANOVA and the Tukey test (**0.001 ≤ p < 0.01, ***p < 0.001).

Three different covering methods (i.e. only plastic lid and the plastic lid with an adhesive or an aluminium foil), and two different medium volumes (1 mL and 2 mL in 24-well plates) were analysed regarding their impact on the medium concentrations after 4 h and 24 h of exposure for three chemicals differing in their volatility (Fig. 2). For the non-volatile difenoconazole (Fig. 2A), there was no significant difference neither between covering methods for both tested time points nor between 1 and 2 mL of exposure medium (p > 0.39, two-way ANOVA). For the semi-volatile DTBP (Fig. 2B), significant chemical losses from the medium were observed, especially after 24 h exposure but these were much more severe when the plates were covered with an adhesive foil than using only the plastic lid followed by the aluminium foil cover with plastic lid. As well, the plate lid had a greater impact than using 1 mL or 2 mL of medium volume although the losses were more severe with the former. For the most volatile chemical, naphthalene (Fig. 2C), losses were the highest. In fact, only with the aluminium cover and a 2 mL volume was naphthalene still well detectable after 24 h. Therefore, the results support the notion that the covering method plays a significant role in chemical loss from the system (Fig. 2), and explain why neither Armitage nor Fischer model were successful in predicting medium concentrations of volatile chemicals (Table 1). Consequently, within this study, empirical models were developed for predicting concentrations of non-volatile and volatile chemicals in exposure media of the plates with different types of covers.

### Empirical model to predict chemical losses

**Figure 3:**
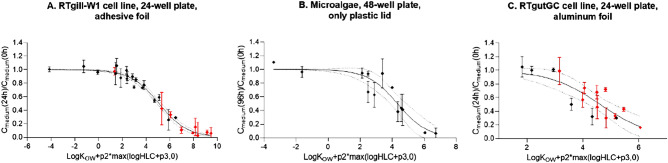
Model calibration (95%CI—confidence intervals) for different covering methods: (**A**) adhesive foil, (**B**) only plastic lid, (**C**) aluminium lid. Chemical concentrations (fraction of concentration in exposure medium at 24 h over 0 h) are plotted dependent on log K_OW_, logHLC and fitted model parameters p2 and p3 from Eq. (3) (for parameter values see Table S5). Red symbols represent volatile chemicals (logHLC > − 5.61^13^).

Given the apparent dependence of the chemical quantity in the medium on the chemicals’ physico-chemical properties and the covering method, the experimental data presented in Table S2 were used in order to calibrate the respective chemical loss models developed in this study (Fig. 3; see also Eq. (3) and model criteria presented in “Materials and methods”).

The models were successfully fitted to the calibration data from all three selected studies: Tanneberger et al.^13^ (R^2^ = 0.98, RMSE = 0.055, Fig. 3A), Dupraz et al.^21^ (R^2^ = 0.86, RMSE = 0.12, Fig. 3B) and Schug et al.^22^ (R^2^ = 0.73 (0.85 without 2 non-volatile chemicals outside of 95% CI), RMSE = 0.15, Fig. 3C). Values of the obtained parameters are presented in Table S5. Table S5 shows that the confidence intervals of p2 and p3 parameters were extremely wide for the data in Fig. 3B, indicating that the chemical volatility did not play a significant role for the data presented there. This outcome was expected because the data behind this figure (i.e. from Dupraz et al.) consisted of only non-volatile chemicals. In addition, although much narrower, the confidence intervals of p2 and p3 parameters for Fig. 3C also question the significance of volatility for the cover with an aluminium foil, as the p2 parameter is not significantly different from zero (Table S5). This finding is in agreement with the results from Fig. 2 indicating that the use of an aluminium foil reduced the impact of volatility on the chemical loss. Consequently, the volatility was clearly most important for the adhesive foil as a cover (narrow and not crossing zero confidence intervals of p2 and p3), which is, at the same time, the most commonly used. Thus, the model for an adhesive foil was validated and further extrapolated based on additional data sets.

### Validation, cross-validation and final calibration of the model for adhesive foil

**Figure 4:**
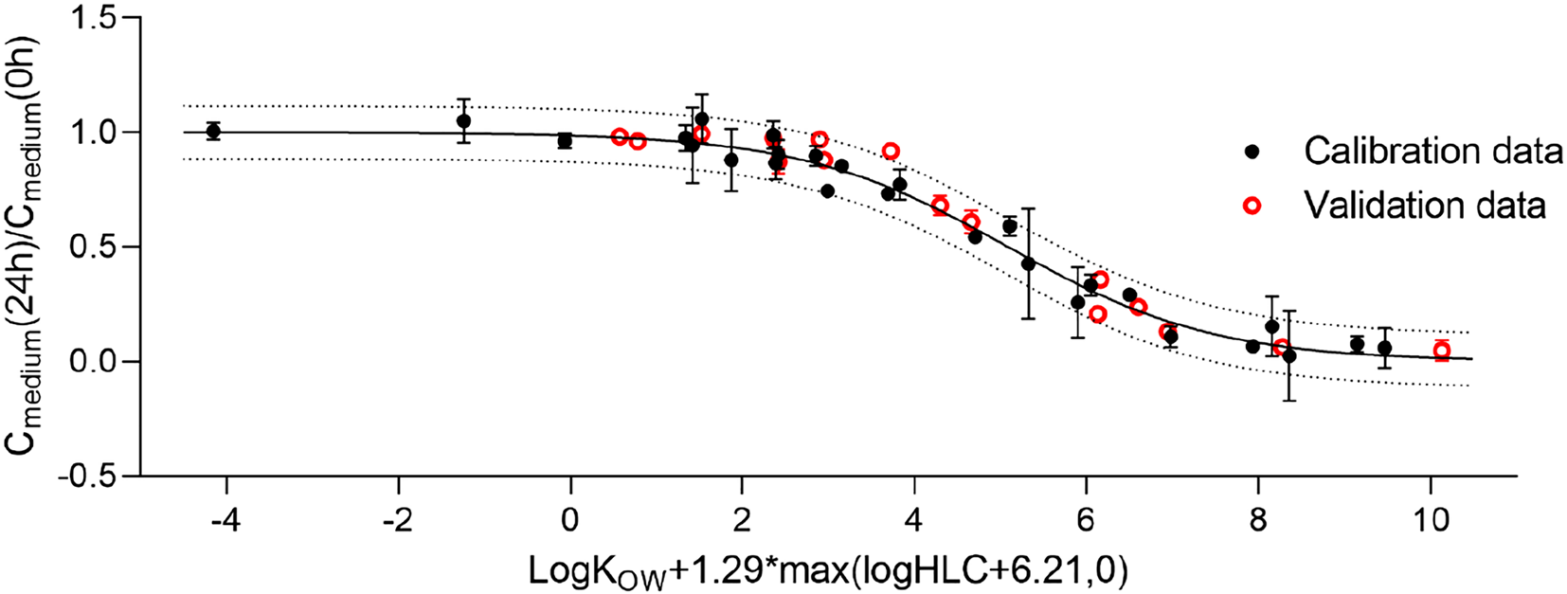
Validation of the empirical model for an adhesive foil covering the 24-well plates containing RTgill-W1 cells. The ratio of measured chemical concentration in the medium after 24 h of exposure and at the beginning of the experiment is plotted versus chemical log K_OW_ and logHLC.

The model for adhesive foil, calibrated based on Tanneberger et al.^13^ data (Figs. 3A and S4), was validated based on fifteen chemicals not used for the model calibration and received from various sources (see “Materials and methods”, “Cross-validation” and Table S3). All the measured medium concentrations used for model validation were within the model 95% prediction bounds (Fig. 4).

For cross-validation (fivefold, 100,000 runs), the repeated data selection, model calibration and validation provided very good results (the mean R^2^ = 0.98, SSE = 0.10 and RMSE = 0.06). In average, 93% of all measured values were within the prediction bounds in all 100,000 model runs with different calibration and validation groups. This value is very high considering the data resolution in which one data point outside the 95% prediction bounds represents 12% of the validation data. In addition, 72.8% of all data outside the prediction bounds were represented by two chemicals: propiconazole (36.4%) and 3,4-dichloroaniline (also 36.4%). In 19.8% of 100,000 model simulations, a higher (propiconazole) or a lower (3–4-dichloroaniline) concentration was measured after 24 h than it would be expected based on the modelling approach (i.e. outside of the prediction bounds).

For the final model calibration, all the data used in the cross-validation test were employed. The results of the final model calibration are presented in Fig. S5. The R^2^ and RMSE values are very similar to those obtained during the cross-validation test. The fitted parameters (Table S6) are not significantly different (95% CI overlap) from those obtained by using only the calibration data (Table S5, Fig. 3A); however, the 95% CI are now narrower. Such obtained final version of the model was then used to extrapolate to other experimental systems as described in “Materials and methods”, “Model extrapolation to other experimental set-ups”.

### Extrapolation of the model for adhesive foil to other plates and biological entities

**Figure 5:**
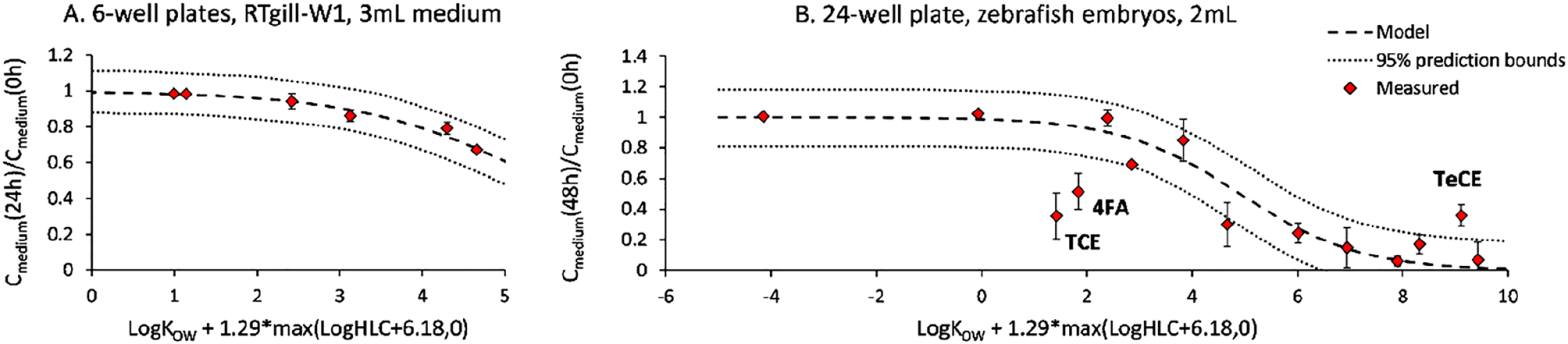
Model extrapolation to other systems covered with an adhesive foil. (**A**) Extrapolation to 6-well plates with RTgill-W1 cell line and medium containing 5% FBS. (**B**) Extrapolation to FET system^12^. Measured ratio of the chemical concentration in the medium at the end and the beginning of the experiment is plotted versus chemical log K_OW_ and logHLC. Abbreviations stand for chemicals of which concentrations were not within the 95% prediction bounds: TCE is 2,2,2-trichloroethanol, 4FA is 4-fluoroaniline, and TeCE is tetrachloroethylene.

In order to extrapolate the model to other small-scale systems, the composition of the well plate formats (i.e. plastic surface, medium and headspace volumes and the biological material content) had to be determined. The chemical partitioning between different compartments of the well were obtained from the Fischer’s partitioning to plastic^18^, our previous work^20^ and this study (see e.g., Figs. S2 and S3), and were included in the model extrapolation (Table S4). First, the model was extrapolated to 6-well plates with RTgill-W1 cell line monolayer (Fig. 5A). Here, all the chemical concentrations were predicted within 95% prediction bounds even though the experimental data came from exposures containing 5% serum (FBS), while the model was calibrated for the serum-free exposure medium. This finding could be accounted for by the fact that we present the chemical concentration at time t as the fraction of the chemical concentration at the beginning of the experiment in the exposure medium which, in this case, also included serum. Next, the model was extrapolated to conditions used in the Fish Embryo Toxicity (FET) test with zebrafish embryos^9^ (Fig. 5B). In general, the model was in a good agreement with measured data. However, it overestimated the concentrations of two chemicals (2,2,2-trichloroethanol and 4-fluoroaniline) and underestimated the concentration of tetrachloroethylene—measured values for these chemicals were outside of the model 95% prediction bounds.

Parameter values for the extrapolated models are presented in Table S7.

### Model applicability for improving toxicity predictions by accounting for chemical losses

In order to test whether the newly developed empirical models can help to improve the assessment of effective concentrations in the absence of chemical analysis, the model for an adhesive foil was applied for two toxicity data sets. In the first, the effective concentrations (EC50s) determined experimentally with the RTgill-W1 cell line for 27 organic chemicals in Tanneberger et al.^13^ were repeated in the same experimental set-up but with 1 mL of exposure medium instead of 2 mL used in Tanneberger et al. The second data set was published by Natsch et al.^29^ and included the comparison between fish LC50 data of several fragrances with the respective measured in vitro (RTgill-W1) EC50s.

**Figure 6:**
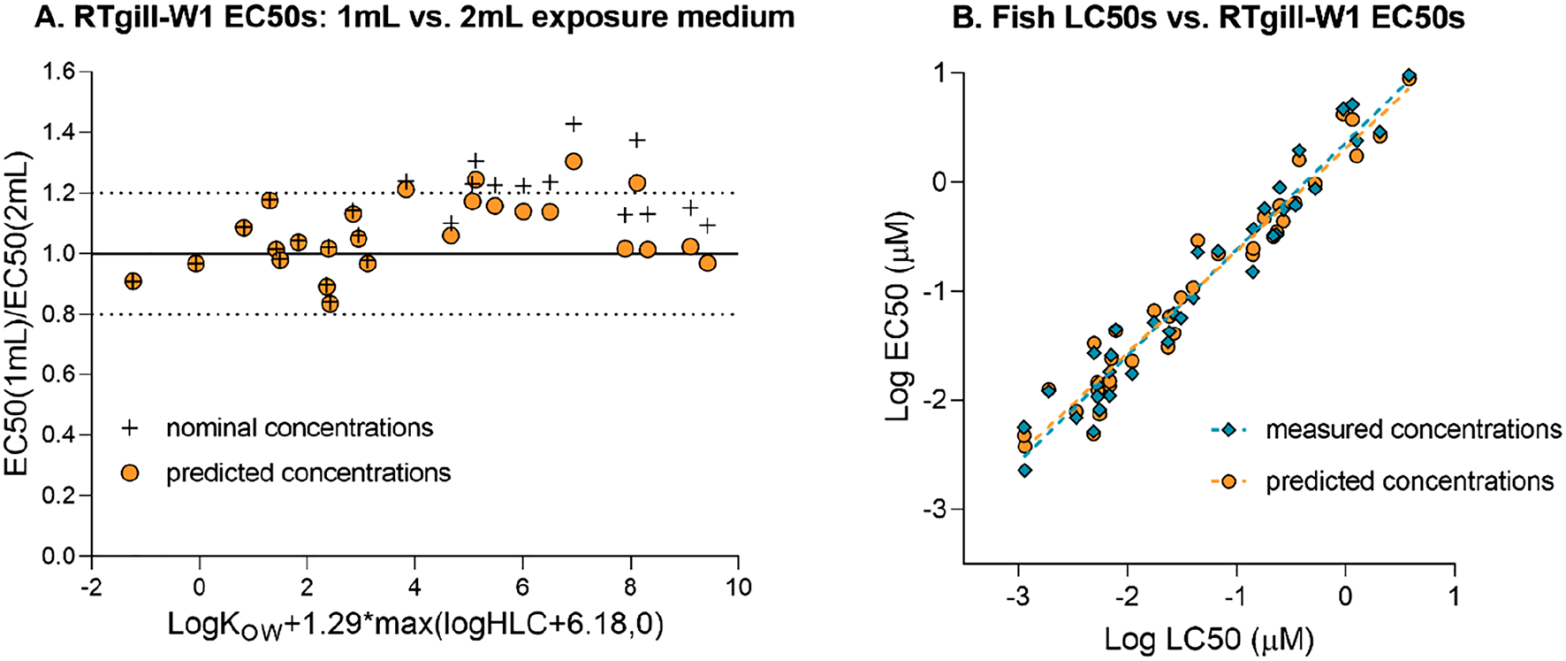
Model applicability for toxicity data. (**A**) Impact of medium volume (1 mL vs. 2 mL) on the difference between the EC50(1 mL) and EC50(2 mL) when nominal or predicted concentrations are used; model parameter values for 1 mL medium are given in Table S7. (**B**) The comparison of fish LC50s with respective cell line EC50s when measured or predicted concentrations are used.

In the first scenario, when nominal concentrations were used and EC50 values obtained with 1 mL and 2 mL medium compared, the volume of exposure medium gained in importance for more hydrophobic and/or volatile chemicals (i.e. x-axis > 3.5, Fig. 6A: circles). In addition, more than 30% of chemicals were characterised by EC50 values that were more than 20% higher (i.e. less toxic) if 1 mL of the exposure medium was used instead of 2 mL. Therefore, our model was applied to predict the concentration-corrected EC50s as described in “Materials and methods” (Fig. 6A: diamonds). As a result, now more than 96% of EC50 values obtained with 1 mL medium were within the 20% difference from the respective EC50 obtained with 2 mL medium. Furthermore, the correlation between the EC50(1 mL)/EC50(2 mL) ratio and the physical–chemical properties of the chemicals, that existed when using nominal concentrations (p < 0.001, F-test), disappeared (p > 0.1, F-test).

Regarding the correlation between fish LC50s and respective cell line EC50s, Fig. 6B confirms that the predicted chemical concentrations were in a very good agreement with the respective measured arithmetic means of concentrations, resulting in the same R^2^ = 0.95 for the LC50 and EC50 comparison. The differences between LC50 values measured for fish and respective EC50s determined with the RTgill-W1 cell line for nominal, measured and predicted chemical concentrations in exposure medium are presented in Table 2.

**Table 2:** Percentage of chemicals of which EC50 values were within the x-fold difference from the measured respective fish LC50s depending on the type of the medium concentrations considered.

x-fold	% of chemicals within x-fold depending on applied concentrations		
	Nominal	Measured^a^	Predicted^b^
Tenfold	94.6	100.0	100.0
Fivefold	83.8	97.3	97.3
Threefold	37.8	70.3	78.4

Fish LC50 and EC50 measured and nominal values were taken from Natsch et al.^29^.

^a^EC50s were determined based on the geometric mean of the concentrations measured at the beginning and the end of the experiment.

^b^EC50s were determined based on the geometric mean of the nominal concentration and the concentration predicted at the end of the experiment.

## Discussion

In comparison to previous approaches (Fig. 1), our developed framework (Figs. 3 and 4) was the only one that successfully predicted medium concentrations of both non-volatile and volatile chemicals, regardless of the applied covering method. In the case of both the Armitage and the Fischer approach, the lack of predictive power may partly result from their assumption that the in vitro systems are fully closed, which they are not, a fact that can be expected to be especially relevant for volatile chemicals. In addition, the Armitage model does not account for chemical binding to the vessel walls or adhesive foil, both processes that can impact measured results^19^. Several prior studies have shown that significant sorption of hydrophobic chemicals to the plastic of microtiter plates takes place, particularly in the absence of serum^3,34,35^, which was the situation applied in the studies to test the Armitage and the Fischer model here. The Fischer approach could be applied successfully to the non-volatile chemicals of these prior experimental studies; indeed, this model showed a good agreement with the measured values of non-volatile chemicals from Tanneberger et al.^13^ (R^2^ = 0.78) and Dupraz et al.^21^ (R^2^ = 0.85). Yet, the finding that it overestimated concentrations in most cases when 24-well plates were applied (Fig. 1A) is likely due to the lack of taking the presence of biological material into account. Adding the lipid content of cells (5%) to the Fischer model did not improve the predictions (R^2^ = 0.72, RMSE = 0.15). However, it is probable that combining Fischer’s approach with more complex and more accurate existing methods for the cellular uptake (e.g.,^15,20,36^) could result in a better agreement with the measured values for non-volatile chemicals than shown in Fig. 1A. For aluminium foil (Fig. 1C) neither Armitage’s nor Fischer’s model predicted the measured concentrations well (R^2^ for both models < 0.7); however, it is important to note that most of the chemicals tested with aluminium foil by Schug et al.^22^ were volatile.

The comparison between Fig. 1A and C indicates that the application of the aluminium foil resulted in lower chemical loss in comparison to the adhesive foil. Indeed, data shown in Fig. 2 confirms that the selection of the covering method is important as soon as more volatile chemicals are being tested. For the semi-volatile and very hydrophobic DTBP, applying either aluminium foil or only the plastic lid resulted in much higher medium concentrations than the one obtained with the adhesive foil. This result is in agreement with the findings by Schreiber et al.^19^ who noticed that the adhesive foil acted as sink and consequently lowered the concentration of phenanthrene and phenanthridine in the exposure medium. Moreover, application of the aluminium foil was the only method that resulted in a measurable medium concentration after 24 h of exposure of the very volatile naphthalene.

The importance of the covers can also be explained with the results of the parameter calibration from Eq. (3) that differ for the system with an adhesive foil and the systems with an aluminium foil or with only the plastic lid. Namely, for the latter ones the p2 parameters were not significantly different from zero and p3 parameters were characterized by very wide confidence intervals, which is in agreement with our hypothesis that volatility did not play a significant role for these two set-ups. Indeed, the model calibration for 24-well plates with the RTgutGC cell line, and covered with an aluminium foil without any glue, indicates that the chemical distribution in such system is different (Fig. 3C). Namely, if the Eq. (3) part describing the chemical volatility (i.e. – p2*max(logHLC + p3,0) was removed, the R^2^ would almost be the same (i.e., 0.83 in comparison to 0.85 from Fig. 3C). Similar results were obtained when the model was fitted to data from Dupraz et al.^21^ that consisted of experiments in 48-well plates with microalgae and covered with the plate plastic lid (R^2^ = 0.86, Fig. 3B). On the other hand, even though the plastic lid or aluminium covers could reduce the impact of volatility on chemical medium concentrations, it is worth noting that applying plate plastic lids as the only cover resulted in high variability of measured chemical concentrations (Fig. 1B). For our three tested chemicals, such variability between the independent replicates was the highest when the aluminium foil was used (Fig. 2). Small amounts of naphthalene were also found in the control wells of the aluminium foil and only plastic lid systems, suggesting some naphthalene carry-over between wells, as also noted by Schug et al.^22^ for two very volatile chemicals (pamplewood and veloutone). Thus, we resonate with the suggestion by Schug et al. that a potential future development could be the use of aluminium foil with a sealing mechanism for each well.

Given that prediction models developed prior to our study did not perform well for volatile chemicals and adhesive foil, along with the fact that adhesive foil is among the most commonly used plate covers, we focussed on developing an empirical model that could account for these experimental settings. Calibrated based on Tanneberger et al.^13^ data and validated based on different independent datasets, the chemical concentrations predicted by our approach were in very good agreement with the concentrations measured in the exposure medium of 24-well plates after 24 h of exposure (Fig. 4). The cross-validation test confirmed the robustness of the model; two chemicals essentially accounted for the concentrations that were outside of the model 95% prediction intervals in 19.8% of 100,000 model simulations: propiconazole and 3,4-dichloroaniline.

The concentration of propiconazole, a non-volatile hydrophobic compound (logK_OW_ = 3.72), was overestimated. It was one of few C^14^ radiolabelled chemicals for which the concentration of the parent form was not separated from its potential biotransformation products^37^. Indeed, fish cell lines can biotransform chemicals^38,39^ and propiconazole is known to be biotransformed in rainbow trout^40^. Thus, it is conceivable that measuring the parent compound only could yield lower concentrations and therefore a better agreement with predictions.

The concentration of 3,4-dichloroaniline, a neither particularly hydrophobic nor volatile chemical (logK_OW_ = 2.69, log HLC = − 5.98), was overestimated by the model. We suspect that this finding might be attributed to the variability in recovery of this compound at termination of exposure in the calibration study^13^ (varying from 63 to 81% of the measured starting concentration). Indeed, when the 3,4-dichloroaniline concentration from the recent Fischer et al.^41^ study, with up to 18% higher recovery, or the Fischer et al. and the Tanneberger et al. reports combined are used instead, this chemical no longer falls outside of the 95% prediction bonds.

The very good results of the model cross-validation gave impetus to further increase the power of the model by including all available data points in the final model calibration (Fig. S5, Table S6). The values of fitted parameters suggest that the volatile chemicals are those that are characterized with logHLC ≥ − 6.18 (i.e. = p3). This value is similar to that of water (log HLC = − 6.38), and, at the same time, significantly lower than the volatility threshold reported previously (i.e. logHLC = − 5.61)^13^. In addition, the fact that p2 parameter from Eq. (3) is higher than one (p2 = 1.29) indicates that for volatile chemicals (i.e. logHLC > − 6.18), their logHLC plays a more important role in the chemical disappearance from the exposure medium than their logK_OW_ values.

The model extrapolation to 6-well plates with RTgill-W1 cell line was successful though based on only a small data set (Fig. 5A). In addition, only one compound tested here was more volatile than water (3,4-dichloroaniline: logK_OW_ = 2.69, logHLC = − 5.98). Therefore, more data are needed to test the model accuracy for volatile chemicals in 6-well plates.

Model extrapolation to the FET system with zebrafish embryos, relying on a larger data set, was likewise successful for the majority of chemicals (Fig. 5B). Yet, three exceptions were noted: the concentrations of two chemicals (4FA and TCE) were overestimated while the concentration of one (TeCE) was underestimated by the model. Concentrations of all these chemicals were, on the other hand, accurately predicted in the system with the RTgill-W1 cell line pointing to peculiarities for these chemicals in the embryo test. One aspect could again be chemical recovery. Indeed, the recovery of TeCE in the embryo system was greater than for the RTgill-W1 cells though only one independent replicate was measured in the embryo (Knöbel et al.^12^) compared to two for the gill cells (Tanneberger et al.^13^). Along these lines, TCE recovery was greater in the RTgill-W1 cells (n = 3) compared to the embryo test (n = 2). Yet, such comparisons lend no support to explain the apparent overestimation for 4FA. Thus, another possibility could be that in embryos, the internal concentrations of TeCE are much lower, and of 4FA and TCE much higher than in fish cells, and as a result, more of TeCE (and less of 4FA and TCE) is measured in the exposure medium than expected. To verify this assumption, we have compared the EC50 values for zebrafish embryos^12^ with the respective values for RTgill-W1 cell line^13^. As no target-specific mechanism of action is assumed for these chemicals (TCE and TeCE are non-polar narcotics and 4FA is a reactive compound^13^), the main differences in their toxicity to cells and embryos could result from the toxicokinetic processes. Indeed, 4FA was five times more toxic and TeCE was 6.5 times less toxic for embryos than for cells, which corresponds well with the differences between their modelled and measured medium concentrations.

The comparison of EC50 values between zebrafish embryos and RTgill-W1 could not explain the differences in the medium concentrations of TCE. We have noted, however, that the ratio between EC50 values based on measured concentrations at the beginning of the experiment and the EC50 based on the nominal concentrations (i.e. EC50(0 h)/EC50(nominal)) was 0.86 for cells, and only 0.47 for embryos. In addition, except for the very volatile naphthalene and instable rotenone, TCE was the only chemical with the main stock recovery, determined at the beginning of the experiment with zebrafish embryos, lower than 60% of the nominal concentration. Thus, probably an unknown process, that influenced the TCE concentrations in the medium, occurred during the experiments with the embryos.

Our model was able to explain the differences between nominal EC50 values obtained with different medium volumes (Fig. 6A). Namely, by using the medium concentration predicted by the model for 24 h exposure together with the nominal starting concentrations to calculate the geometric mean, apparent differences in EC50 values for 1 mL or 2 mL exposure volume disappeared. In addition, the EC50(1 mL)/EC50(2 mL) ratio was no longer correlated with the physical–chemical properties of the chemicals, even for very hydrophobic and volatile chemicals. These results confirm that the differences between the EC50s determined when dissimilar volumes of exposure medium (and headspace) were used, were caused by the chemical distribution rather than by different toxicological mechanisms in the in vitro system, and that our model has accurately quantified these differences.

By again predicting the medium concentrations at termination of exposure, our model moreover supported a very similar in vitro–in vivo correlation compared to when measured exposure concentrations were used (Fig. 6B). In addition, Table 2 shows that almost 80% of EC50s determined by Natsch et al.^29^ were within threefold difference from respective fish LC50s when the model-based predicted concentrations were applied, in contrast to less than 40% if only nominal concentrations would have been used. Therefore, in the experimental set-up described by Natsch et al.^29^, the chemical concentrations predicted by our model could be used as substitutes of measured concentrations.

In summary, our approach can successfully be used for predicting concentrations of non-volatile and volatile chemicals in exposure medium of well-plate systems. In addition, the results for zebrafish embryos (48 h experiment duration) and microalgae (96 h experiment duration) indicate that the applicability of our framework reaches beyond the in vitro systems with fish cell lines (24 h) for which it was primarily intended. Potentially, it can be calibrated with any specific set-up; in case of unavailable measured data, it could be extrapolated by using the knowledge of the plate dimensions and the partitioning data from Fischer et al.^18^ and Stadnicka-Michalak et al.^20^. If, however, measured concentrations are available, significant differences between those and the respective modelled values can point out anomalies that could be further investigated. Limitations of the model as it currently stands point toward important future research. One potential limitation is the reliance on volatility and hydrophobicity as determinants of medium concentration, which may, for example, not hold up well for chemicals that show a strong pH-dependent partitioning behaviour or preferentially bind to cellular proteins. As well, our model accounts for chemical distribution in the absence of serum or other protein- and lipid rich medium components, where one can assume that the predicted medium concentration is approximating the freely bioavailable chemical concentration. In the presence of complex medium supplements, however, much less of the chemical may be freely bioavailable. There is great potential to combining our empirical approach with other complementary methods, such as mechanistic approaches that describe the chemical binding to serum components^15,41^, or kinetic models to learn about the chemicals’ internal concentrations^20^. Therefore, our framework is an important building block to significantly improve the applicability of various small-scale systems. Prior to doing an experiment, it can help identify if sufficient chemical is expected to be left in the medium at the end of the exposure period or if special precautions are needed to prevent such unacceptable losses. For interpretation of an experiment, it provides accurate chemical medium concentrations without resource- and time-intensive analytical measurements.

## Supplementary Information

Supplementary Information.
