# Supplementary material for: Predicting exposure concentrations of chemicals with a wide range of volatility and hydrophobicity in different multi-well plate set-ups
Source: Sci Rep. 2021 Feb 25;11:4680. doi: 10.1038/s41598-021-84109-9 (PMC7907087; doi:10.1038/s41598-021-84109-9)
Supplement: Supplementary file 1 — Supplementary Information. [file 41598_2021_84109_MOESM1_ESM.docx]

**SUPPORTING INFORMATION (SI)**

**Predicting exposure concentrations of chemicals with a wide range of volatility and hydrophobicity in different multi-well plate set-ups**

Julita Stadnicka-Michalak^1,2^, Nadine Bramaz^1^, René Schönenberger^1^ and Kristin Schirmer^1,2,3^

*1 Eawag, Swiss Federal Institute of Aquatic Science and Technology, 8600 Dübendorf, Switzerland. 2 EPF Lausanne, School of Architecture, Civil and Environmental Engineering, 1015 Lausanne, Switzerland. 3 ETH Zürich, Department of Environmental Systems Science, 8092 Zürich, Switzerland*

Table of Contents

**Quality check of measured data - data points excluded from the analysis2**

**Experiments with different covers - chemical analytics 3**

**References13**

**Table S1.** Values of the input parameters for 24-well plates with cell monolayer and for 48-well plate with microalgae**5**

**Table S2.** Physico-chemical properties of chemicals used to test modelling approaches **5**

**Table S3.** Chemical properties and experimental set-ups of data used for the model validation**7**

**Table S4.** Well parameters of different in vitro systems**8**

**Table S5.** Values of the parameters fitted to eq. 1 – calibration data (with 95%CI)**8**

**Table S6.** Final model calibration for an adhesive foil - values of the parameters fitted to eq. 1**8**

**Table S7.** Parameters (with CI95) of the extrapolated models presented in Figures 5 and 6**9**

**Figure S1.** Schematic representation of the study design**10**

**Figure S2.** Possible chemical distribution in a plastic well **11**

**Figure S3.** Chemical partitioning between cells and plastic surface in 24-well plate**11**

**Figure S4.** Calibration of the empirical model for an adhesive foil**12**

**Figure S5.** Final model calibration based on all available data for an adhesive foil cover**12**

**Quality check of measured data - data points excluded from the analysis**

Only chemicals with measured medium concentrations were considered. From Schug et al.^1^, ratios (and their standard deviations) of measured concentrations at the end and at the beginning of the experiments were taken directly from the supplementary table provided by Schug et al., while for Tanneberger et al.^2^ and Dupraz et al.^3^ such ratios were calculated in our study based on provided raw data.

From the data set presented in Tanneberger et al.^2^, two chemicals have been excluded: menadione and dichlorophen. Menadione, a synthetic vitamin K3, is known to be very unstable in the presence of trace minerals^4^ which was the case in cytotoxicity experiments performed by Tanneberger et al.^2^ Dichlorophen, on the other hand, is known for undergoing photodegradation processes^5^, and therefore the potential degradation of menadione and dichlorophen in the exposure medium could not be solely attributed to its LogK_OW_ and logHLC properties. The instability of these two chemicals in the dosing stocks of performed cytotoxicity experiments supports this assumption. Thus, in case of the lack of measured chemical concentrations, the knowledge of the chemical of interest might be crucial for accurate predictions of its behaviour in the well-plate system.

For the purpose of our study, the ratios between the chemical medium concentration at the end of the experiment (i.e. 24h) and at the beginning of the experiment (i.e. 0h) was determined to quantify the chemical losses from the exposure media at the end of the experiments. Such ratios were calculated for all six chemical concentrations and their average was used. In few cases, the ratio for the highest or the lowest tested concentration differed significantly from the ratios of five other chemical concentrations. As this was most probably due to the concentrations causing the death and detachment of cells (high concentrations) or the concentrations around the analytical limits of quantification (low concentrations), the outlying ratios were deleted for such cases.

Data set from Dupraz et al.^3^ included measured concentrations of 17 chemicals. Measurements of one chemical, flazasulfuron, were characterised by very high variability (chemical medium concentrations recovered at the end of experiments varied between 12 and 72% of the concentrations measured at time point 0h), and therefore were excluded from the analysis. In addition, data were excluded when the chemical concentrations measured at the end of experiments were more than 20% higher than the concentrations measured at the beginning of the experiment. As only one data point (i.e. for one concentration and one replicate) was available for each of such chemicals, it was not possible to verify why the final concentrations were much higher than the starting ones (case for three chemicals: diuron, S-metolachlor and imidacloprid)

**Experiments with different covers - chemical analysis**

Concentrations of chemicals (nominal concentration = 1μM) used for testing different covering methods were determined with a High Pressure Liquid Chromatograph equipped with a Fluorescence Detector (HP1200, HPLC-FLD, Agilent Technologies, Waldbronn, Germany) with the following procedure:

- For non-volatile difenoconazole:
  - At each time point 900μL of L15/ex medium containing a chemical (or a control sample) was taken from each of the three wells (i.e. technical replicates) and put into the centrifuge tube into which 900μL of MTBE was added.
  - Samples were vortexed for 2min, then centrifuged for 5min at 14,000 rpm at 4°C. 800µl from the supernatant was transferred to an HPLC vial.
  - Samples were measured with the Macherey Nagel AG, EC100/2 Nucleodur 100-3, C18ec, Cat. 760054.20 HPLC column.
  - Used eluents: 99.95% nanopure water & 0.05% formic acid and 99.95% Acetonitril & 0.05% formic acid
  - The detector used was DAD at 240nm and the injection volume was 40μL at 6°C.
- For semi-volatile DTBP and volatile naphthalene:
  - 900μL of L15/ex medium containing a chemical (or a control sample) was taken from each of the three wells (i.e. technical replicates), transferred into the HPLC vial and then directly measured. 0h samples were stored overnight at -80^o^C.
  - Samples were measured with the Macherey Nagel AG, EC100/2 Nucleodur 100-3, C18ec, Cat. 760054.20 HPLC column.
  - Used eluents: 100% nanopure water and 100% methanol
  - The detector used was FLD with excitation/emission wavelength being 224 nm and 330 nm, respectively; the injection volume was 25μL at 6^o^C.

**Table S1.** Values of the input parameters for 24-well plates with cell monolayer and for 48-well plate with microalgae (i.e. systems used for testing the Armitage model^6^).

| **Input parameter** | **24-well plate with cells** | **48-well plate with microalgae** |
| --- | --- | --- |
| Total vessel volume (mL) | 3.3 | 1.7 |
| Bulk medium volume (mL) | 2 | 1 |
| Chemical concentration (M) | Chemical dependent | Chemical dependent |
| Cell total lipid content (-) | 0.05^a^ | 0.05^a^ |
| Cell total protein content (-) | 0.055^b^ | 0.05^a^ |
| Cell density (kg/L) | 1 | 1 |
| Total mass of cells (mg) | 0.157 | 0.1 |
| Temperature (^o^C) | 19 | 20 |
| Serum fraction (-) | 0 | 0 |
| Serum composition | Not applicable | Not applicable |

^a^Values set for the model consideration of the biological material

^b^Cell total protein content was calculated as an average of internally measured proteins of RTgill-W1, RTL-W1 and RTgutGC cell lines

**Table S2.** Physico-chemical properties of chemicals used to test modelling approaches.

| **Chemical** | **LogK_OW_**  **[-]** | **LogHLC**  **[aꞏm**^3^**/mol]** | **LogK_AW_**  **[-]** | **MW [g/mol]** | **MP**  **[^o^C]** | **C_SAT,W_ [mg/L]** | **C_24h_/C_0h_^a^**  **(SD), [-]** |
| --- | --- | --- | --- | --- | --- | --- | --- |
| Data from Tanneberger et al.^12^ , 24-well PS plates, RTgill-W1, adhesive foil cover, 24h exposure | | | | | | | |
| 2,4-dinitrophenol | -1.24 | -7.56 | -5.45 | 184.1 | 118.5 | 2.79E+03 | 1.05 (0.038) |
| Caffeine | -0.07 | -10.45 | -8.83 | 194.2 | 238.0 | 2.16E+04 | 0.96 (0.095) |
| allyl alcohol | 0.17 | -5.30 | -3.69 | 58.1 | -129.0 | 1.00E+06 | 0.98 (0.033) |
| aniline | 0.9 | -5.72 | -4.08 | 93.1 | -6.0 | 3.60E+04 | 1.06 (0.057) |
| 4-fluoroaniline | 1.15 | -5.65 | -4.04 | 111.1 | -0.8 | 1.12E+04 | 0.88 (0.108) |
| dichloromethane | 1.25 | -2.49 | -0.88 | 84.9 | -95.1 | 1.30E+04 | 0.33 (0.047) |
| 2,2,2-trichloroethanol | 1.42 | -6.81 | -5.20 | 149.4 | 19.0 | 8.33E+04 | 0.94 (0.045) |
| N-methylaniline | 1.66 | -5.05 | -3.44 | 107.2 | -57.0 | 5.62E+03 | 0.85 (0.028) |
| Malathion | 2.36 | -9.08 | -6.70 | 330.4 | 2.8 | 1.43E+02 | 0.99 (0.057) |
| 4-chlorophenol | 2.39 | -6.38 | -4.59 | 128.6 | 42.7 | 2.40E+04 | 0.87 (0.06) |
| diethylphthalate | 2.42 | -6.40 | -4.60 | 222.2 | -40.5 | 1.08E+03 | 0.91 (0.072) |
| 3,4-dichloroaniline | 2.69 | -5.98 | -3.22 | 162.0 | 46.2 | 9.20E+01 | 0.74 (0.024) |
| 2,4,6-trichlorphenol | 2.85 | -6.64 | -3.97 | 197.5 | 69.0 | 8.00E+02 | 0.90 (0.040) |
| 2,3-dimethyl-1,3-butadiene | 3.13 | -1.30 | 0.31 | 82.2 | -76.0 | 3.26E+02 | 0.06 (0.043) |
| naphthalene | 3.3 | -3.36 | -1.75 | 128.2 | 80.2 | 3.10E+01 | 0.11 (0.086) |
| tetrachloroethylene | 3.4 | -1.75 | -0.14 | 165.8 | -22.3 | 2.06E+02 | 0.08 (0.046) |
| 1,2-dichlorobenzene | 3.43 | -2.72 | -1.10 | 147.0 | -16.7 | 8.00E+01 | 0.07 (0.035) |
| parathion ethyl | 3.83 | -6.53 | -4.91 | 291.3 | 6.1 | 1.10E+01 | 0.77 (0.019) |
| disulfoton | 4.02 | -5.68 | -4.05 | 274.4 | -25.0 | 1.63E+01 | 0.54 (0.067) |
| 1,2,4-trichlorobenzene | 4.02 | -2.85 | -1.24 | 181.5 | 17.0 | 4.90E+01 | 0.03 (0.025) |
| lindane | 4.14 | -5.29 | -3.68 | 290.8 | 112.5 | 8.00E+00 | 0.43 (0.195) |
| pentachlorophenol | 3.69^b^ | -6.90 | -6.00 | 266.3 | 174.0 | 1.40E+01 | 0.73 (0.015) |
| di-n-butylphthalate | 4.5 | -5.74 | -10.45 | 406.9 | 166.5 | 1.40E+02 | 0.59 (0.195) |
| hexachlorophene | 5.9^c^ | -12.07 | -10.45 | 406.9 | 166.5 | 1.40E+02 | 0.26 (0.043) |
| 4-decylaniline | 6.04 | -4.57 | -2.96 | 233.4 | 103.5 | 2.01E-01 | 0.15 (0.153) |
| permethrin | 6.5 | -6.54 | -4.12 | 391.3 | 34.0 | 6.00E-03 | 0.29 (0.129) |
| hexamethylenetetramine | -4.15 | -8.78 | -7.17 | 140.2 | 65.1 | 4.49E+05 | 1.01 (0.013) |
| Data from Dupraz et al.^20^, 48-well PS plates, microalgae, plastic lid cover, 96h exposure | | | | | | | |
| isoproturon | 2.5 | -4.84 | -8.34 | 206.3 | 108.8 | 6.50E+01 | 0.94 (0.035) |
| fipronil | 3.75 | -3.64 | -7.46 | 437.2 | 203.9 | 1.90E+00 | 0.74 (-) |
| metazachlor | 2.13 | -4.23 | -7.64 | 277.8 | 164.1 | 6.71E+02 | 0.95 (0.075) |
| glyphosate | -3.2 | -6.68 | -10.07 | 169.1 | 204.2 | 1.05E+04 | 1.11 (-) |
| AMPA | -1.63 | -0.8 | -13.29 | 111.0 | 61.6 | 1.00E+06 | 0.96 (0.081) |
| a-cypermethrin | 5.5 | -1.16 | -4.77 | 416.3 | 82.1 | 1.15E-01 | 0.08 (-) |
| acrinathrin | 5.24 | -1.97 | -6.32 | 541.5 | 81.5 | 2.00E-02 | 0.07 (0.073) |
| chlorpyrifos | 4.7 | -0.32 | -3.92 | 350.6 | 82.9 | 1.12E+00 | 0.44 (0.202) |
| chlorpyrifos-methyl | 4.24 | -0.63 | -4.92 | 258.7 | 123.8 | 3.11E-01 | 0.38 (0.149) |
| azoxystrobin | 2.5 | -8.13 | -11.53 | 403.4 | 228.1 | 6.00E+00 | 0.67 (0.156) |
| kresoxim-methyl | 3.4 | -3.44 | -6.84 | 313.4 | 137.4 | 2.00E+00 | 0.94 (0.214) |
| quinoxyfen | 4.66 | -1.5 | -6.40 | 308.1 | 148.1 | 1.16E-01 | 0.41 (0.230) |
| spiroxamine | 2.89 | -2.42 | -5.36 | 297.5 | 105.8 | 4.70E+02 | 0.63 (0.179) |
| Data from Schug et al.^21^, 24-well PS plates, RTgutGC, aluminum foil cover, 24h exposure | | | | | | | |
| Eugenol F | 1.82 | -6.1 | -7.481 | 164.2 | 60.6 | 1154 | 1.05 (0.15) |
| Methyl-antranilate | 2.17 | -6.1 | -4.11 | 151.2 | 55.8 | 2790 | 1.00 (0.06) |
| Lilyflore® | 2.94 | -6.8 | -4.95 | 176.3 | 71.32 | 609 | 1.01 (0.03) |
| Alpinolide | 5.2 | -6.2 | -4.58^d^ | 282.4 | n/a | 1.94 | 0.30 (0.01) |
| Helvetol | 4.33 | -5.9 | -4.77 | 228.4 | 63.71 | 37.5 | 0.32 (0.12) |
| Pamplewood | 4.37 | -2.3 | n/a | n/a | n/a | n/a | 0.50 (0.09) |
| Veloutone® | 4.6 | -3.1 | n/a | n/a | n/a | n/a | 0.57 (0.11) |
| Verdox® | 4.75 | -2.7 | n/a | n/a | n/a | n/a | 0.74 (0.08) |
| Nirvanol® | 4.99 | -3.7 | n/a | n/a | n/a | n/a | 0.46 (0.17) |
| Cetalox® | 5.09 | -4.6 | -1.70 | 236.4 | 74.13 | 1.9 | 0.72 (0.03) |
| Cachalox® | 5.09 | -3.2 | n/a | n/a | n/a | n/a | 0.68 (0.10) |
| Exaltenone | 5.15 | -4.4 | n/a | n/a | n/a | n/a | 0.30 (0.15) |
| Muscenone® delta | 5.52 | -4.2 | -1.51 | 236.4 | 51.94 | 0.1 | 0.33 (0.04) |
| Tonalide® | 5.7 | -4.8 | -2.25 | 258.4 | 106.87 | 1.2 | 0.43 (0.03) |
| Vulcanolide® | 6.25 | -5 | -2.50 | 258.4 | 105.42 | 0.03 | 0.16 (0.01) |
| Damascone beta | 3.68 | -3.5 | -2.33 | 192.3 | 52.5 | 194 | 0.99 (0.20) |

^a^C_24h_/C_0h_ refers to the ratio of the chemical concentration in the exposure medium measured at the end and at the beginning of the experiment, SD are standard deviations. SD were generally based on five concentrations (i.e. data points). For values without SD, indicated as (-), only one data point was available.

^b^ Pentachlorophenol is a polar chemical that dissociates at the medium’s pH of 7.1; thus, its pH-corrected logK_OW_ was taken from Nowosielski et al.^24^

^c^ Hexachlorophene dissociates at pH of the medium and therefore its logD (for pH = 7.4) taken from ChemSpider was used instead of logK_OW_

^d^ air-water partition coefficient (LogKaw) was calculated based on ideal gas constant, temperature and Henry’s Law constant, as provided in Schug et al.^21^

**Table S3.** Chemical properties and experimental set-ups of data used for the model validation.

| **Chemical** | **CAS** | **LogK_OW_** | **LogHLC^a^** | **Plate** | **Cell line/embryo** |
| --- | --- | --- | --- | --- | --- |
| 2-mercaptobenzothiazole | 149-30-4 | 2.42 | -7.44 | 24-well | no cells^b^/RTgill-W1 |
| naphthalene | 91-20-3 | 3.30 | -3.36 | 24-well | no cells^b^/RTgill-W1 |
| difenoconazole | 119446-68-3 | 4.30 | -11.05 | 24-well | no cells^b^/RTgill-W1 |
| 2,4-di-tert-butylphenol | 96-76-4 | 5.19 | -5.43 | 24-well | no cells^b^/RTgill-W1 |
| 1,2,3-trichlorobenzene | 87-61-6 | 4.05 | -2.9 | 24-well | RTgill-W1 |
| hexachlorobenzene | 118-74-1 | 5.73 | -2.77 | 24-well | RTgill-W1 |
| carbendazim | 10605-21-7 | 1.52 | -10.67 | 24-well | RTgill-W1 |
| cypermethrin | 52315-07-8 | 6.60 | -6.38 | 24-well | RTgill-W1 |
| Dimethoate | 60-51-5 | 0.78 | -9.61 | 24-well | RTgill-W1 |
| Imidacloprid | 138261-41-3 | 0.57 | -14.78 | 24-well | RTgill-W1 |
| Malathion | 121-75-5 | 2.36 | -8.31 | 24-well | RTgill-W1 |
| cyproconazole | 94361-06-5 | 2.9 | -9.15 | 24-well | RTgill-W1 |
| propiconazole | 60207-90-1 | 3.72 | -8.76 | 24-well | RTgill-W1 |
| benzo(a)pyrene^c^ | 50-32-8 | 6.13 | -6.34 | 24-well | RTgill-W1 |
| 2,4-di-tert-butylphenol^c^ | 96-76-4 | 5.19 | -5.43 | 24-well | RTgut-GC |
| 2-mercaptobenzothiazole | 149-30-4 | 2.42 | -7.44 | 6-well^d^ | RTgill-W1 |
| 3,4-dichloroaniline | 95-76-1 | 2.69 | -5.98 | 6-well^d^ | RTgill-W1 |
| difenoconazole | 119446-68-3 | 4.30 | -11.05 | 6-well^d^ | RTgill-W1 |
| topramezone | 210631-68-8 | 1.14 | -19.6 | 6-well^d^ | RTgill-W1 |
| Methimazole | 60-56-0 | -0.34 | -5.69 | 6-well^d^ | RTgill-W1 |
| Quinoxyfen | 124495-18-7 | 4.66 | -6.18 | 6-well^d^ | RTgill-W1 |
| Caffeine | 58-08-2 | -0.07 | -10.45 | 24-well | zebrafish embryo |
| allyl alcohol | 107-18-6 | 0.17 | -5.30 | 24-well | zebrafish embryo |
| 4-fluoroaniline | 371-40-4 | 1.15 | -5.65 | 24-well | zebrafish embryo |
| dichloromethane | 75-09-2 | 1.25 | -2.49 | 24-well | zebrafish embryo |
| 2,2,2-trichloroethanol | 115-20-8 | 1.42 | -6.81 | 24-well | zebrafish embryo |
| 4-chlorophenol | 106-48-9 | 2.39 | -6.38 | 24-well | zebrafish embryo |
| 2,4,6-trichlorphenol | 88-06-2 | 2.85 | -6.64 | 24-well | zebrafish embryo |
| 2,3-dimethyl-1,3-butadiene | 513-81-5 | 3.13 | -1.30 | 24-well | zebrafish embryo |
| naphthalene | 91-20-3 | 3.30 | -3.36 | 24-well | zebrafish embryo |
| tetrachloroethylene | 127-18-4 | 3.40 | -1.75 | 24-well | zebrafish embryo |
| 1,2-dichlorobenzene | 95-50-1 | 3.43 | -2.72 | 24-well | zebrafish embryo |
| parathion ethyl | 56-38-2 | 3.83 | -6.53 | 24-well | zebrafish embryo |
| disulfoton | 298-04-4 | 4.02 | -5.68 | 24-well | zebrafish embryo |
| 1,2,4-trichlorobenzene | 120-82-1 | 4.02 | -2.85 | 24-well | zebrafish embryo |
| hexamethylenetetramine | 100-97-0 | -4.15 | -8.78 | 24-well | zebrafish embryo |

log K_OW_ and HLC values were taken from EPISuite database (experimental value if available). In addition, they were corrected based on available pKa values

^a^ Henry’s Law constant is given in units atm·m^3^·mol^-1^

^b^ Experiments were carried out in L15/ex medium as well as in L15 medium + 5% FBS

^c^ Experiment was carried out in L15 medium + 5% FBS

^d^ Experiments in 6-well plates were carried out with 3mL of L15 medium containing 5% of FBS

**Table S4.** Well parameters of different in vitro systems.

| **Parameter** | **24-well plate - RTgill-W1 (default) or embryos** | | | **6-well plate** |
| --- | --- | --- | --- | --- |
|  | 2mL medium | 1mL medium | Embryos - 2mL |  |
| Radius^a^, (R1+R2)/2 (cm^2^) | 0.800 | 0.800 | 0.800 | 1.750 |
| Well height, H_w_ (cm) | 1.650 | 1.650 | 1.650 | 1.900 |
| Well volume, (cm^3^) | 3.313 | 3.313 | 3.313 | 18.28 |
| Well surface, (cm^2^) | 10.23 | 10.23 | 10.23 | 24.39 |
| Well lateral surface, (cm^2^) | 8.291 | 8.291 | 8.291 | 20.89 |
| Medium height, H_m_ (cm) | 0.996 | 0.498 | 0.996 | 0.311 |
| Well lateral surface touching the medium, (cm^2^) | 5.005 | 2.502 | 6.941 | 3.428 |
| Headspace volume, (cm^3^) | 1.313 | 2.313 | 1.313 | 15.38 |
| Medium volume, (cm^3^) | 2.000 | 1.000 | 2.000 | 3.000 |
| Surface of cell monolayer or embryo (cm^2^) | 1.936 | 1.936 | 1.539 ꞏ 10^-2^ | 3.5 |
| Volume of one cell or embryo (cm^3^) | 1.767 ꞏ 10^-9^ | 1.767 ꞏ 10^-9^ | 1.796 ꞏ 10^-4^ | 1.767 ꞏ 10^-9^ |

**Table S5.** Values of the parameters fitted to eq. 1 – calibration data (with 95%CI).

| f(logK_OW_, logHLC) = 1/(1+10^((p1-logK_OW_-p2*max(logHLC+p3,0))*p4)) | | | |
| --- | --- | --- | --- |
| Coefficient | Fig. 3A (adhesive foil) | Fig. 3B (PS plastic lid) | Fig. 3C (aluminium foil) |
| p1 | 5.09 (4.74, 5.43) | 4.34 (3.455, 5.22) | 4.62 (3.33, 5.916) |
| p2 | 1.29 (1.05, 1.51) | -0.92 (±inf.)* | -0.218 (-0.7289, 0.2939) |
| p3 | 6.21 (5.82, 6.61) | 5.95 (±inf.)* | 5.9 (-1.877, 13.68) |
| p4 | -0.362 (-0.424, -0.301) | -0.467 (-0.7576, -0.1769) | -0.473(-0.8045, -0.1407) |

*Extremely wide confidence intervals indicate that in this case the volatility does not play a role in the model, which was expected as the data from Fig 3B did not include any volatile chemical

**Table S6.** Final model calibration for an adhesive foil with values of the parameters fitted to eq. 1.

| f(logK_OW_, logHLC) = 1/(1+10^((p1-logK_OW_-p2*max(logHLC+p3,0))*p4)) | | |
| --- | --- | --- |
| Coefficient | Value | CI95% |
| p1 | 5.15 | (4.92, 5.38) |
| p2 | 1.29 | (1.10, 1.47) |
| p3 | 6.18 | (5.91, 6.46) |
| p4 | -0.39 | (-0.441, -0.339) |

**Table S7.** Parameters (with CI95) of the extrapolated models presented in Figures 5 and 6.

| f(logK_OW_, logHLC) = 1/(1+10^((p1-logK_OW_-p2*max(logHLC+p3,0))*p4)) | | | |
| --- | --- | --- | --- |
| Coefficient | Figure 5A | Figure 5B | Figure 6A |
| p1 | 5.48 (5.25, 5.71) | 4.91 (4.68, 5.14) | 4.81 (4.60, 5.02) |
| p2 | 1.29 (1.10, 1.47) | 1.29 (1.10, 1.47) | 1.29 (1.10, 1.47) |
| p3 | 6.18 (5.91, 6.46) | 6.18 (5.91, 6.46) | 6.18 (5.91, 6.46) |
| p4 | -0.393 (-0.435, -0.335) | -0.384 (-0.439, -0.342) | -0.39 (-0.435, -0.335) |


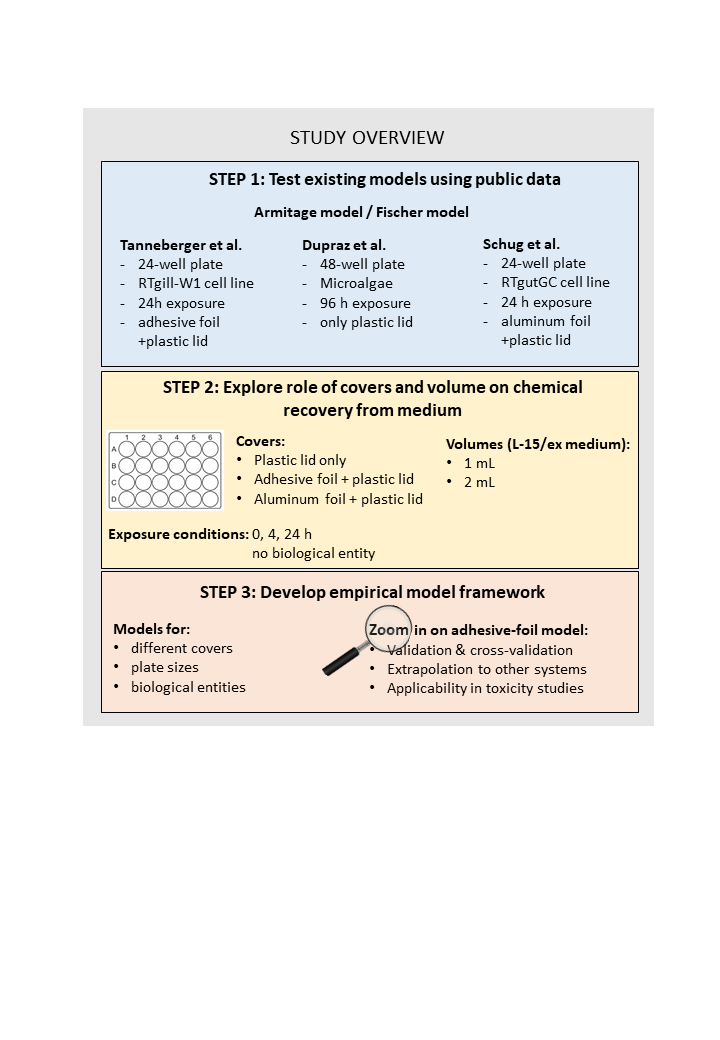


***Figure S1.*** *Schematic representation of the study design. Existing models for predicting chemical losses were developed by Armitage et al.^13^ and Fischer et al. ^17^. Measured data used for testing existing approaches and calibration of empirical models come from Tanneberger et al.^12^, Dupraz et al.^20^ and Schug et al.^21^*


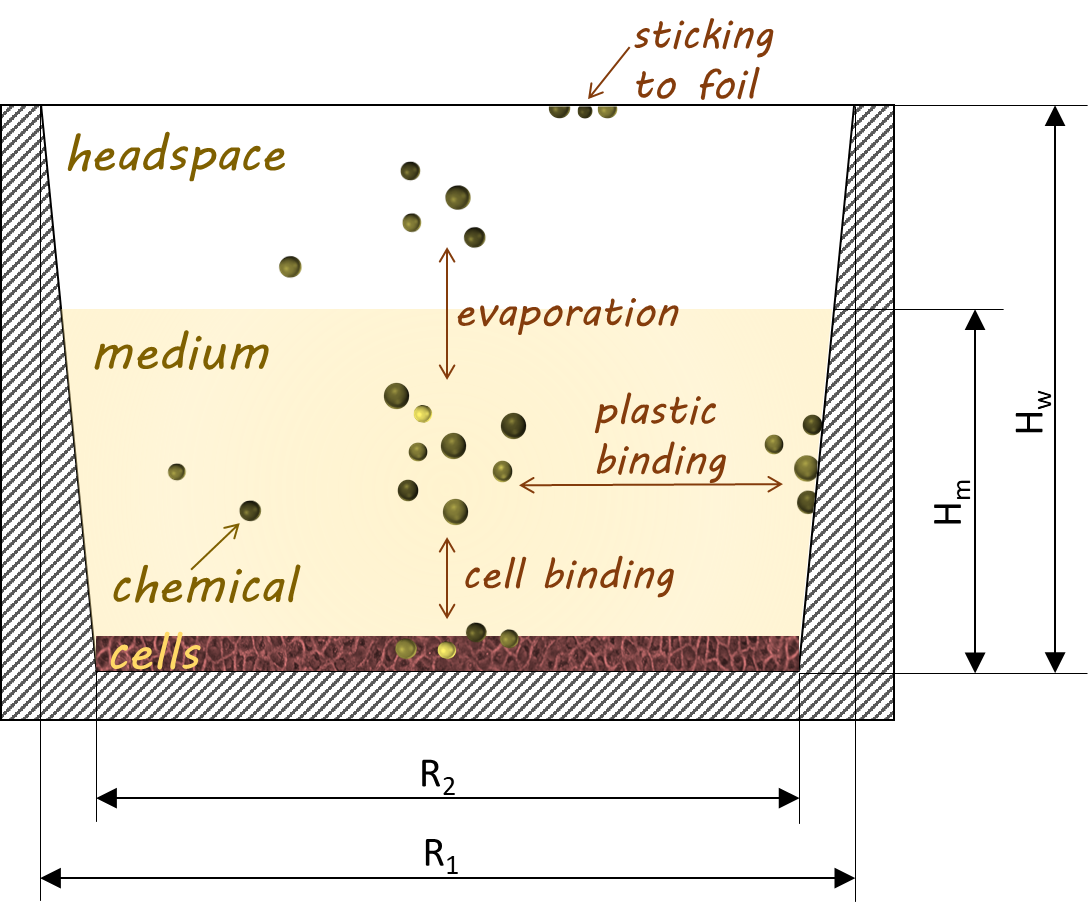

$$V_{w}=\frac{\pi}{3}H_{w}\left( {R_{1}}^{2}+R_{1}R_{2}+{R_{2}}^{2} \right)$$

***Figure S2.*** *Possible chemical distribution in a plastic well. Values of the symbols are presented in Table S4.*


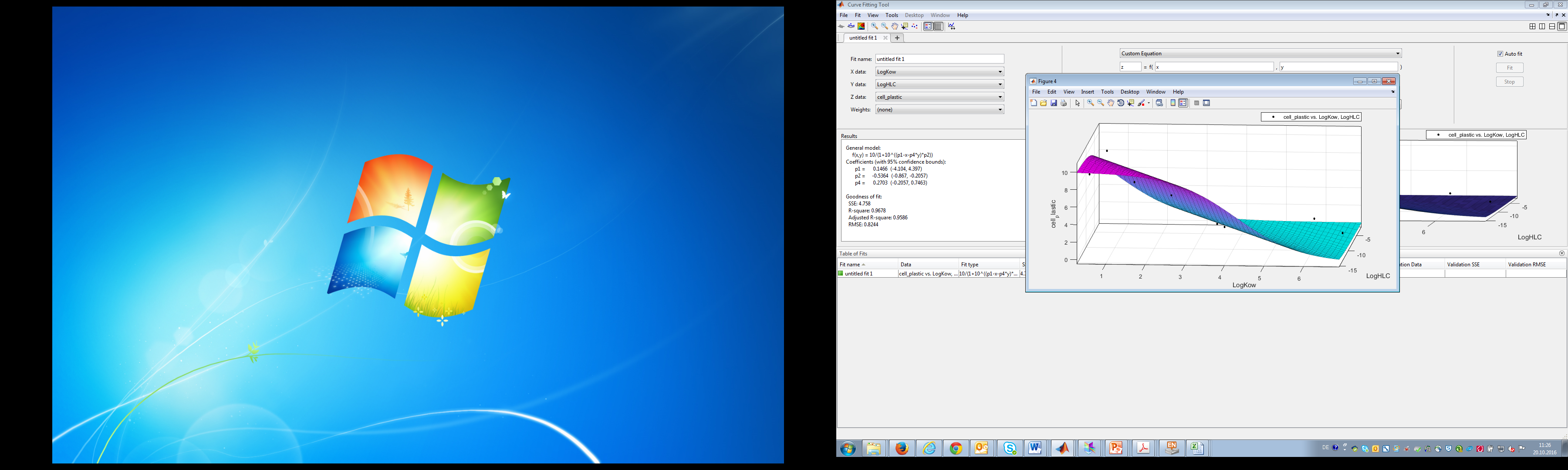


K_cell-plastic_

***Figure S3****. Chemical partitioning between cells and plastic surface in 24-well plate depending on LogK_OW_ and LogHLC – measured data taken from Stadnicka-Michalak et al.^7^*


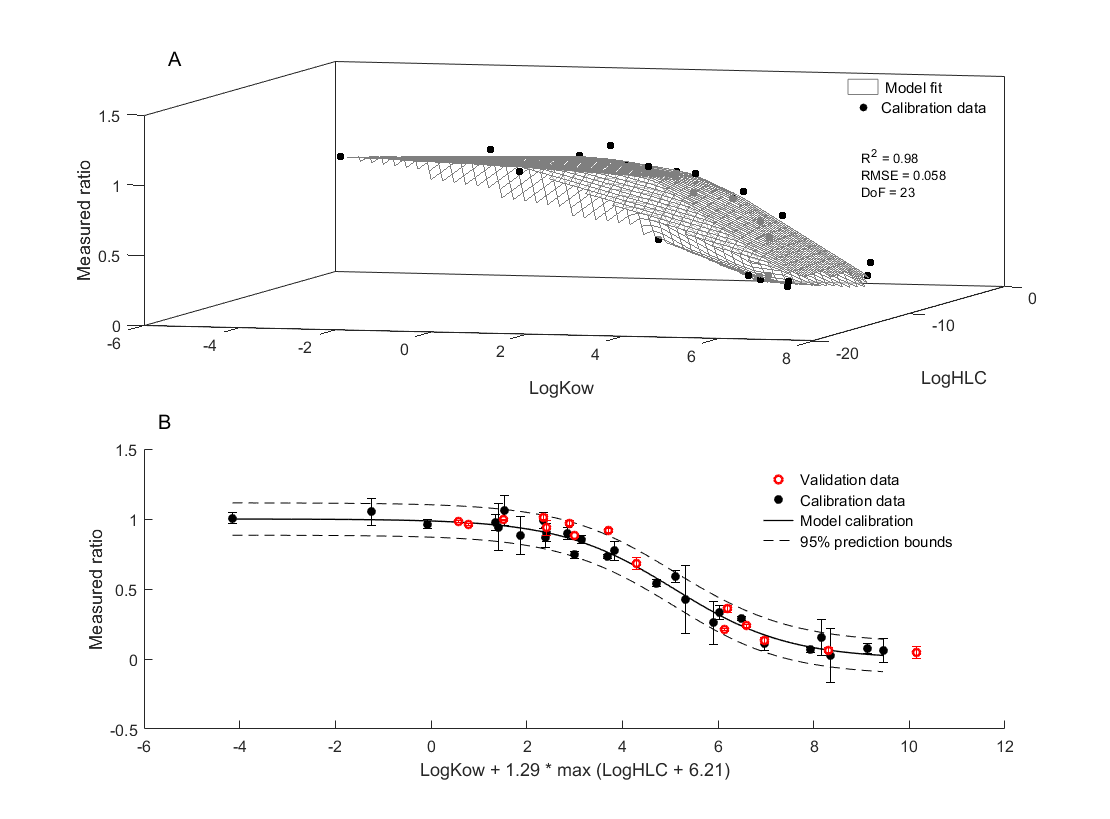


**Figure S4.** Calibration of the empirical model for an adhesive foil covering the 24-well plates containing RTgill-W1 cells. Ratio of the measured chemical concentration in the medium after 24h of exposure and at the beginning of the experiment is plotted versus chemical log K_OW_ and logHLC. It is a different representation of the Fig. 3A from the main manuscript.


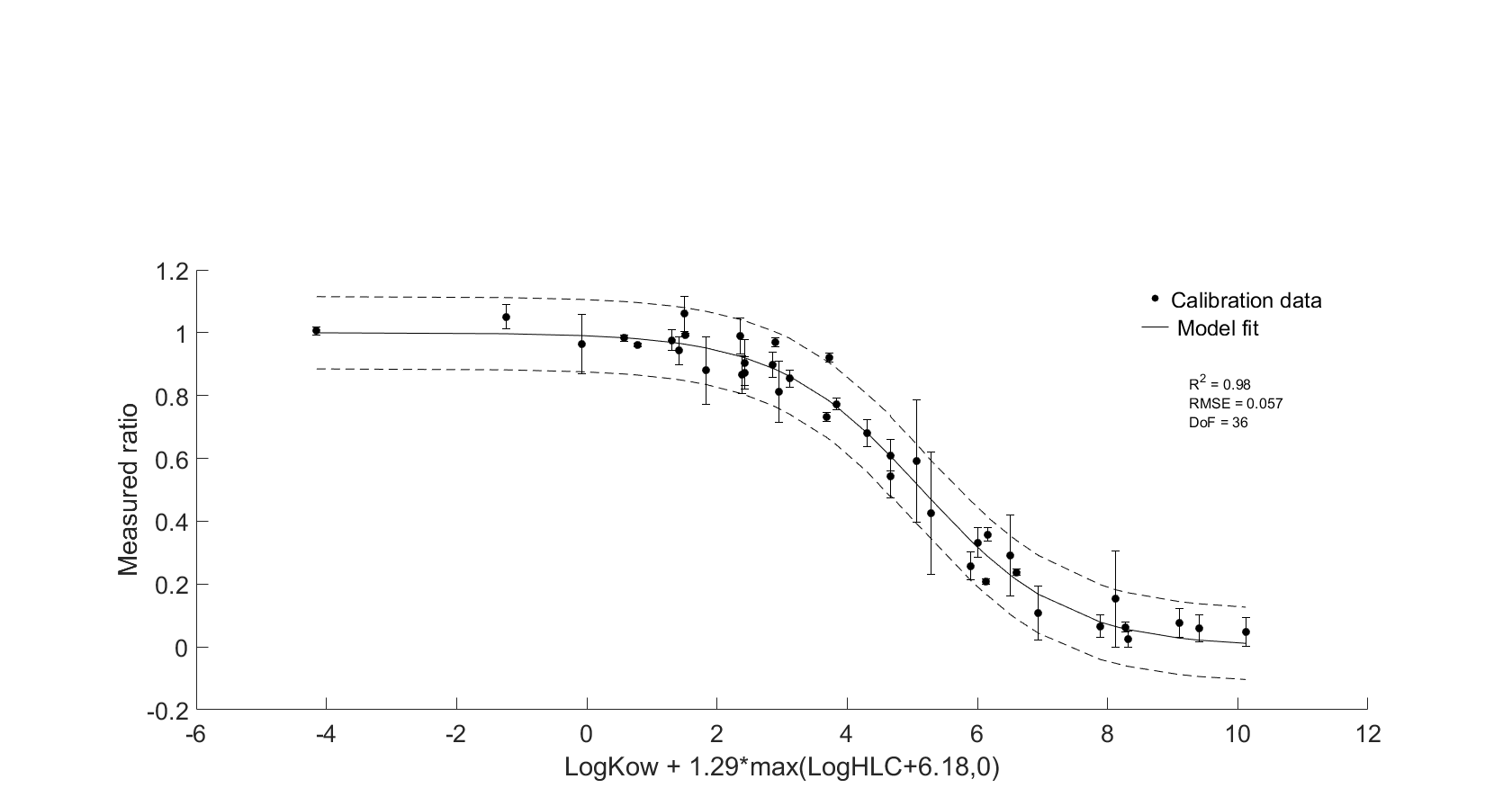


**Figure S5.** Final model calibration based on all available data for an adhesive foil cover. Ratio of the measured chemical concentration in the medium after 24h of exposure and at the beginning of the experiment is plotted versus chemical log K_OW_ and logHLC.

**References**

1 Schug, H. *et al.* Extending the concept of predicting fish acute toxicity in vitro to the intestinal cell line RTgutGC. *ALTEX - Alternatives to animal experimentation*, doi:10.14573/altex.1905032 (2019).

2 Tanneberger, K. *et al.* Predicting Fish Acute Toxicity Using a Fish Gill Cell Line-Based Toxicity Assay. *Environ. Sci. Technol.* **47**, 1110-1119, doi:10.1021/es303505z (2013).

3 Dupraz, V. *et al.* Demonstrating the need for chemical exposure characterisation in a microplate test system: toxicity screening of sixteen pesticides on two marine microalgae. *Chemosphere* **221**, 278-291, doi:<https://doi.org/10.1016/j.chemosphere.2019.01.035> (2019).

4 Frye, T. M. in *Vitamin Nutrition Update-Seminar Series 2* p. 70 (Hoffmann-La Roche, Inc., 1978).

5 Zertal, A., Jacquet, M., Lavédrine, B. & Sehili, T. Photodegradation of chlorinated pesticides dispersed on sand. *Chemosphere* **58**, 1431-1437, doi:<https://doi.org/10.1016/j.chemosphere.2004.09.085> (2005).

6 Armitage, J. M., Wania, F. & Arnot, J. A. Application of Mass Balance Models and the Chemical Activity Concept To Facilitate the Use of in Vitro Toxicity Data for Risk Assessment. *Environ. Sci. Technol.* **48**, 9770-9779, doi:10.1021/es501955g (2014).

7 Stadnicka-Michalak, J., Tanneberger, K., Schirmer, K. & Ashauer, R. Measured and modeled toxicokinetics in cultured fish cells and application to *in vitro – in vivo* toxicity extrapolation. *PLoS ONE* **9**, e92303 (2014).
